# Geno-pheno characterization of crop rhizospheres: an integrated Raman spectroscopy and microbiome approach in conventional and organic agriculture

**DOI:** 10.3389/fmicb.2025.1721013

**Published:** 2025-11-28

**Authors:** Yejin Son, Peisheng He, Mathew Baldwin, Guangyu Li, Zijian Wang, April Z. Gu, Jenny Kao-Kniffin

**Affiliations:** 1 School of Integrative Plant Science, Cornell University, Ithaca, NY, United States; 2 School of Civil and Environmental Engineering, Cornell University, Ithaca, NY, United States; 3 School of Biological and Environmental Engineering, Cornell University, Ithaca, NY, United States

**Keywords:** soil microorganisms, organic agriculture, conventional agriculture, agricultural microbiome, Raman single cell spectroscopy, food security

## Abstract

**Introduction:**

Agricultural management practices strongly influence soil microbiomes, with broad implications for ecosystem function. Yet, the combined phenotypic and compositional dynamics of rhizosphere microbial communities across conventional and organic farming systems remain poorly characterized, underscoring the need for integrated approaches to understand how management decisions drive microbial assembly and function.

**Methods:**

We investigated microbial communities associated with conventionally and organically cultivated horticultural crops across multiple farms in New York State. To capture both taxonomic and functional dimensions, community composition was characterized using 16S rRNA gene sequencing, and phenotypic traits were assessed with a newly developed single-cell Raman microspectroscopy (SCRS) approach. This dual strategy allowed us to link microbial identity with metabolic potential and adaptive traits.

**Results:**

Farming practice significantly shaped microbiome clustering, independent of site or plant species. SCRS-based phenotyping revealed distinct biochemical profiles: organic systems favored lipid-accumulating phenotypes linked to energy storage and stress resilience, whereas conventional systems promoted carbon-rich phenotypes associated with rapid assimilation and biomass production. Network analysis identified Pseudomonas and nitrogen-fixing taxa as ecological hubs in conventional systems, while organic soils were enriched in Bacilli class plant growth-promoting rhizobacteria (e.g., *Tumebacillus, Bacillus, Paenibacillus, Brevibacillus*) and contained microorganisms bearing antibiotic resistance genes.

**Discussion:**

Our findings highlighted that management regimes drive distinct microbial functional traits and community structures. By integrating genotypic and phenotypic analyses, particularly microbial phenotyping via SCRS, we uncovered adaptive traits that differentiate conventional and organic systems, offering new insight into how plant production practices shape microbial assembly and ecological function.

## 1 Introduction

The Green Revolution of the 1960s helped boost agricultural productivity with the widespread use of synthetic fertilizers, pesticides, and fuels to maximize crop yields globally. Decades after, conventional agricultural practices became widespread, but many have raised significant environmental concerns, including soil erosion, water pollution, pesticide overreliance, and biodiversity loss (Mäder et al., 2002; Tilman et al., 2002; Gabriel et al., 2013; Hartmann et al., 2015; Lori et al., 2017; Lupatini et al., 2017; Azarbad, 2022). In contrast to conventionally managed systems, organic agriculture has emerged to limit synthetic fertilizer and pesticide use while promoting soil health practices for long-term sustainability (Mäder et al., 2002; Gabriel et al., 2013; Hartmann et al., 2015; Lupatini et al., 2017). Although organic systems often yield less and with greater variability than conventional agriculture, they consistently provide ecological advantages, including enhanced biodiversity, improved soil carbon sequestration, and competitive profitability driven by reduced input costs and market premiums (Mäder et al., 2002; Smith et al., 2019). Thus, organic agriculture as a form of low-input food production enables the conservation of soil, water, energy, and biodiversity to benefit long-term sustainable food production (Gabriel et al., 2013).

Understanding how different agricultural systems shape soil microbiomes is essential for evaluating their long-term effects on ecosystem resilience, nutrient cycling, and sustainable food production. Soil microorganisms play a vital role in agricultural productivity by forming complex networks of mutualistic, competitive, deleterious, and neutral interactions within interconnected ecological communities (Shade et al., 2012; Toju et al., 2018). A community of microorganisms (microbiome) in a given agricultural soil habitat determines soil ecosystem functions as a whole, extensively mediating soil nutrient cycling and nutrient supply for plants, soil moisture control, organic matter formation, soil remediation, and plant pest and disease suppression (Hartmann et al., 2015; Lori et al., 2017; King et al., 2022). Thus, comparing soil microbiomes between conventional and organic systems can reveal how farming practices influence microbial diversity, soil health, and nutrient cycling, informing sustainable agricultural strategies that balance productivity with ecosystem resilience.

Conventional and organic agricultural practices can influence soil microbial community composition and function due to their distinctive impacts on the soil environment. Many attempts have been made to compare soil microbiome characteristics between conventional and organic agriculture. Previous studies, spanning both short-term and long-term investigations, have documented significant differences in microbial community structure and functional traits between conventional and organic agricultural systems (Hartmann et al., 2015; Pershina et al., 2015; Lupatini et al., 2017; Wang et al., 2017; Chen et al., 2020; Peltoniemi et al., 2021; Jung et al., 2024), but some sites showed only a minor difference in terms of microbial composition (Pershina et al., 2015). In general, the distinctions between conventional and organic agriculture can arise from the differential use of agrochemicals such as chemical fertilizers and pesticides. A review of research suggested that the overuse of pesticides had negative effects on soil microbial communities that in the worst case, led to significant yield loss, decreased soil microbial biomass, and reduced soil respiration (Sharma et al., 2019). In contrast, organic agricultural soils are known for having greater microbial diversity and taxonomic heterogeneity compared to conventionally managed fields with crop rotations of wheat, barley, potato, lily, carrot, and maize (Lupatini et al., 2017) while Wang et al. (2017) observed an increase in bacterial richness under organic systems. These findings suggest the potential benefits of organic farming in maintaining soil microbial diversity, which can contribute to improved ecosystem stability, nutrient cycling, and long-term agricultural sustainability.

Among the various approaches for comparing microbiome variations, single-cell Raman spectroscopy (SCRS) has recently emerged as a promising method for microbial characterization. While well-developed and widely applied method to analyze taxonomic composition of bacterial communities, such as 16S rRNA gene sequencing, have been used to compare genotypic variations, phenotyping the rhizomicrobiome has rarely been explored due to the lack of suitable techniques with sufficient resolution (Liu et al., 2023). Existing approaches in phenotyping the rhizomicrobiome either rely on specific biomarkers, such as metabolomics coupled with stable isotopes in tracing rhizomicrobial pathways (Achouak and Haichar, 2019), and/or only measure in bulk, such as enzymatic activity assays which only reflect the collective phenotypic profile of the rhizomicrobiome (Bai et al., 2013). The SCRS, as a label-free phenotyping method, offers a non-invasive, high-resolution metabolic fingerprinting of the microbiome that reflects the cellular composition and proteomics-like metabolics state of microorganisms in the soil ecosystem (Hermans et al., 2023). The SCRS captures cell-specific metabolic fingerprints without the need for cultivation or labeling, enabling high-resolution phenotyping of individual microorganisms within their native soil matrix. This is particularly critical in rhizomicrobiome studies, where SCRS enables high-resolution profiling of soil microbial functions by capturing cell-specific metabolic fingerprints that uncover functional diversity, ecological roles, and microbe–plant interactions often obscured by bulk analyses. Previously, few applications of SCRS in environmental microbial analysis were mostly restricted within simpler matrices such as highly engineered enhanced biological phosphorus removal (EBPR) systems (Majed et al., 2012; Li et al., 2018), and/or by the reliance on labeled biomarkers such as the use of stable isotope probing in nitrogen fixation activity (Li et al., 2018). To date, only a few attempts in characterizing specific groups of the microbes from the rhizomicrobiome with SCRS have been documented (Li et al., 2018; He et al., 2025).

However, many comparative studies on soil microbiomes are constrained to one or more plant species or typically limited to comparisons within a single site, making it difficult to identify broad, overarching patterns across conventional and organic farming systems. For instance, many comparative studies on soil microbiomes in conventional and organic agriculture limited the number of sites and plant species analyzed to reduce confounding spatial, temporal, and plant variability (Hartmann et al., 2015; Pershina et al., 2015; Bonanomi et al., 2016; Lupatini et al., 2017; Wang et al., 2017; Kundel et al., 2020; Peltoniemi et al., 2021; Jung et al., 2024), with the exception of a few large-scale surveys, such as the study of Chen et al. (2020) that examined diverse vegetable plants grown in 30 conventional and organic greenhouses across China. While some research reported clear distinctions in microbial composition and biodiversity metrics (Lupatini et al., 2017; Wang et al., 2017), both Chen et al. (2020) and Jung et al. (2024) detected no significant differences in alpha diversity of soil microbiomes between conventional and organic agricultural systems for greenhouse vegetables such as tomato, cucumber, eggplant, green pepper and rice. The lack of consistent differences in biodiversity across studies suggests that broader environmental variables may play a significant role in shaping soil microbial communities. Possibly, variability in soil biogeography, temperature, microclimatic factors, plants, and on-farm practices, likely contribute to challenges in interpreting impacts of conventional and organic agriculture on soil microbial diversity (Hartmann et al., 2015; Geisen, 2021; Hermans et al., 2023). To address these limitations, large-scale microbiome surveys incorporating diverse crops and geographic regions are essential for identifying robust and generalizable trends in microbial dynamics across conventional and organic farming systems.

Most comparative studies on soil microbiomes are constrained to one or more plant species or typically limited to comparisons within a single site, making it difficult to identify broad, overarching patterns across conventional and organic farming systems. For instance, many comparative studies on soil microbiomes in conventional and organic agriculture limited the number of sites and plant species analyzed to reduce confounding spatial, temporal, and plant variability (Hartmann et al., 2015; Pershina et al., 2015; Bonanomi et al., 2016; Lupatini et al., 2017; Wang et al., 2017; Kundel et al., 2020; Peltoniemi et al., 2021; Jung et al., 2024), with the exception of a few large-scale surveys, such as the study of Chen et al. (2020) that examined diverse vegetable plants grown in 30 conventional and organic greenhouses across China. To uncover robust and generalizable trends in microbial dynamics between farming systems, there is a pressing need for large-scale microbiome surveys that incorporate a wide range of crops and geographic regions.

In this study, we performed a large-scale comparison of soil microbiomes derived from conventional and organic agricultural systems spanning 22 horticultural crop plants grown across various sites in New York State, USA. Specifically, we examined the soil microbiomes of 10 conventionally grown plants and 12 organically grown plants grown in different farm locations. We characterized the composition of soil prokaryotic microbial communities between conventional and organic agriculture using the 16S rRNA gene sequencing. In complement to the commonly used amplicon sequencing, we employed a SCRS method to pioneer the characterization of phenotypic profiles of the rhizomicrobiome of various crops grown in conventional and organic farming systems, using a similar method to our previous study (He et al., 2025) to enable us to obtain single-cell-resolution phenotypic profiles of rhizomicrobiome in various crops that reveal functional information beyond taxonomic units. Combining this SCRS with amplicon sequencing, we aimed to link phenotypic characteristics with variation in taxonomic composition, resulting in a deeper insight into microbial functions and interactions within conventional and organic farming systems. To summarize, our objectives were to: (1) identify distinct attributes of soil microbial communities in the rhizosphere of 22 different plants grown in conventionally and organically managed agricultural systems, and (2) link the complex microbial structure-phenotype relationships within the rhizomicrobiome under the influence of different agricultural practices.

## 2 Materials and methods

### 2.1 Farm surveys and sample collection

We sampled rhizosphere soils from a total of 12 commonly found and ecologically important crop plants, including potato, tomato, carrot, lettuce, squash, corn, wheat, soybean, alfalfa, triticale, oat, and pea from two conventional farms and two organic farms (Table 1). Supplementary Figure 1 presents a map generated using ArcGIS Pro 3.4 (Esri Inc., USA) and the Imagery Hybrid basemap from ArcGIS Online (Esri Earthstar Geographics the GIS User Community, 2025). It illustrates the spatial distribution of collection sites both within and between farms, highlighting variations in location, soil type, and environmental heterogeneity. The two conventional farms are permitted to apply agrochemicals, including chemical fertilizers, pesticides, and herbicides, while complying with federal and state regulations governing their use (The Environmental Protection Agency, 2025). Both Hellers Farm CSA and Titus Zimmerman's farm utilized inorganic NPK fertilizers; however, Hellers Farm CSA also applied pesticides and herbicides, while Titus Zimmerman's farm adopted enhanced soil management practices such as cover cropping and crop rotation, which is more closely aligned with organic agriculture. In contrast, the two organic farms, which adhered to restrictions on synthetic fertilizers and pesticides while utilizing organic amendments such as animal manures, were certified in compliance with the regulations of the United States Department of Agriculture (United States Department of Agriculture, 2025). Mainstreet farms applied certified organic fertilizers composed of poultry-based farmyard manure and composted plant residues, while Lakeview Organic Grain LLC utilized both poultry- and dairy-based farmyard manures, integrated with cover cropping and crop rotation. In addition, Lakeview applied rock phosphate as a supplemental phosphorus source. This sampling strategy was designed to encompass a broader and more representative range of conventional and organic farming practices, thereby enhancing the ecological scope of our comparative analysis.

**Table 1:** Summary of surveyed crop plants and source farms.

**Production type**	**Crop name**	**Farm details**	**Farm fertilization scheme**	**Growth stage**	**Soil moisture (%)%**
Conventional agriculture	Carrot	Hellers farm CSA (42°6′50.8^′′^N 75°8′37.7^′′^W)	Inorganic NPK fertilizers accompanied with herbicides, insecticides and fungicides	At full maturity, ready for harvest	22.0
	Lettuce				28.4
	Potato				31.1
	Squash				25.5
	Tomato				24.8
	Alfalfa	Titus Zimmerman's farm (42°2′17.3^′′^N 76°9′36.5^′′^W)	Inorganic NPK fertilizers but integrated with cover cropping and diverse crop rotations to enhance overall soil management practices	At flowering stage	25.0
	Corn			At full maturity, ready for harvest	23.6
	Soybean				25.6
	Triticale				23.6
	Wheat				25.4
Organic agriculture	Carrot	Mainstreet farms (42°4′5.0^′′^N 76°1′34.2^′′^W)	Organic fertilizers, including poultry-based farmyard manure and composted materials	At full maturity, ready for harvest	25.3
	Lettuce				22.5
	Potato				28.4
	Squash				24.9
	Tomato				21.7
	Alfalfa			At flowering stage	23.4
	Corn	Lakeview Organic Grain LLC (42°1′34.5^′′^N 76°9′48.0^′′^W)	Organic fertilizers including slow-release poultry- and dairy-based farmyard manure in combination with cover cropping and crop rotation. Rock phosphate used as a supplemental nutrient source	At full maturity, ready for harvest	24.0
	Soybean				23.3
	Triticale				26.3
	Wheat				22.8
	Oat				28.7
	Pea				26

Sampling was performed during the summer of 2021 (June 28^th^ to July 22^nd^) with a local temperature averaged at 20 °C and an average local precipitation of 176.53 mm during this period. Soil moisture in the collected samples ranged from 21.7% to 31.1% (Table 1). At the time of sampling, all surveyed plants had reached full maturity and were ready for harvest, as sampling was aligned with each farm's respective harvest schedule. Consequently, the sampled plants represent the fully developed growth stage of each species. For each crop species, five replicate plants were randomly selected from spatially distributed locations across the field to enhance sample representativeness. Each plant was excavated using a soil auger, and rhizosphere soil was carefully collected from the 0–20 cm depth along with the root system. Samples were stored at 4 °C in ice-filled coolers during transport to the laboratory, where rhizosphere soils were meticulously separated from roots. Soil samples were either processed immediately for SCRS and soil moisture measurements or stored under−80 °C until further analysis. The soil auger was meticulously cleaned and sterilized with 70% ethanol between sampling replicates to prevent cross-contamination.

### 2.2 DNA extraction, PCR amplification, and library preparation

Rhizosphere soil samples were sieved through 2 mm mesh and ground thoroughly before DNA extraction. We used 0.25 g of a soil sample to extract soil DNA using DNeasy PowerSoil Pro Kits (Cat#: 47014; Qiagen, Carlsbad, CA) following the company's manual. The extracted DNA was amplified by primers 515F (5′- GTGYCAGCMGCCGCGGTAA−3′) and 806R (5′- GGACTACNVGGGTWTCTAAT−3′) to target the V4 hypervariable region of bacterial and archaeal 16s rRNA. PCR reactions were conducted using 10 μl of 2 x AccuStart II PCR ToughMix (QuantaBio, Beverly, MA, United States), 1 μl of 10 μM forward and reverse primers with final concentrations of 0.5 μM, 6 μl of DNase-free PCR Water (MoBio, Carlsbad, CA, United States), and 2 μl of sample DNA. The PCR thermocycling conditions were as follows: 94 °C for 2 min; 25 cycles at 94 °C for 20 s, at 55 °C for 20 s, and at 72 °C for 30 s; with a final elongation at 72 °C for 5 min. We used the method introduced in Howard et al. (2017) to prepare sequencing libraries based on unique two-barcode index combinations (Nextera, Illumina). Samples were subsequently normalized in clear 96-well plates prior to sequencing to ensure uniform input concentrations across libraries via SequalPrep™ Normalization Plat (96) kits (Thermo Fisher Scientific, US). The pooled libraries were paired-end sequenced using the NovaSeq 6000 S4 (Illumina, San Diego, CA, USA) at Novogene (Durham, NC).

### 2.3 Bioinformatics

Raw forward and reverse reads were trimmed using Cutadapt to remove adaptor contaminants (Martin, 2011). Forward and reverse reads which had more than Phred offset score 20 were merged to create single consensus sequences using BBMerge (Bushnell et al., 2017) at the default setting. Bioinformatics analysis and classification of microbial communities from sequencing data were conducted following the methods described by He et al. (2025). Amplicon sequence variants (ASVs) were created using DADA2 (Callahan et al., 2016) in the denoise-single mode on the QIIME2 platform (Bolyen et al., 2019). Uchime algorithm was applied to correct for amplicon sequencing errors and identify and remove chimeric reads. ASVs were taxonomically assigned using a Naive-Bayes classifier trained on the Greengenes2 2022.10 database through the q2-feature-classifier plugin (McDonald et al., 2022). The ASV data were transferred to R v.4.3.0 (R Core Team, 2023) and rarefied to the lowest read depth of any sample in the dataset using the function *rarefy_even_depth* in the phyloseq package (McMurdie and Holmes, 2013). The completeness of rarefaction was assessed based on coverage-based rarefaction and extrapolation (R/E) curves with Hill number (q=1, Shannon diversity) using the package iNEXT.3D (Hsieh et al., 2016; Chao et al., 2021, 2023) (Supplementary Figure 2). These curves help estimate how well the observed diversity represents the true diversity by plotting species accumulation as sequencing depth (sample size) increases. The results demonstrated that all samples exceeded 99% coverage after rarefaction, preserving most observed microbial diversity and enabling reliable comparisons across agricultural systems with minimal ecological resolution loss.

### 2.4 Microbiome structure assessments

For rhizosphere microbiome processing, we employed the phyloseq package (McMurdie and Holmes, 2013) in R v.4.3.0 (R Core Team, 2023) to efficiently handle and analyze microbial community data. Initially, the rarified ASV counts were normalized as percentages to facilitate comparisons of relative abundance. ASVs with a mean relative abundance less than 0.5% were subsequently filtered out. Additionally, the dataset was refined by excluding taxa found in fewer than 80% of replicated samples within each plant type group, ensuring a robust ecological representation. The Bray-Curtis dissimilarity matrix was constructed using the function *distance* in the vegan package (Oksanen et al., 2007) and visualized via NMDS with the function *ordinate* in the phyloseq package (McMurdie and Holmes, 2013). The ASV abundance data were transformed into CLR using the microbiome package (Lahthi and Shetty, 2017) before significant variations in beta diversity was assessed using PERMANOVA by employing the function *adonis2* in the vegan package (Oksanen et al., 2007). The CLR transformation helps mitigate mathematical distortions that can occur when proportional microbiome data is directly analyzed with Bray-Curtis or Hellinger methods, ensuring a more accurate representation of microbial community differences (Gloor et al., 2017).

### 2.5 Differential abundance (DA) analysis

We conducted DA analysis based on the non-rarified ASV data, as rarified microbiome data are often prone to increased Type-I and Type-II errors in DA analysis of microbiome data (McMurdie and Holmes, 2013; Nearing et al., 2022). The ANOVA-like differential expression (ALDEx) analysis was chosen for the DA analysis due to its low false discovery rates compared to other DA methods (Ma et al., 2020; Nearing et al., 2022; Yang and Chen, 2022). The ASV counts were CLR-transformed after Monte Carlo samples of Dirichlet distributions were calculated using the package ALDEx2 (v.1.30.0) (Fernandes et al., 2013) in R. Significantly differential taxa among treatments were identified by the BH-adjusted *p* values < 0.05 derived from generalized linear models incorporating Production × Plant species interactions.

### 2.6 Network analysis

Network analyses were applied to construct co-abundance networks separately for rhizomicrobiome samples from conventional and organic systems, in order to identify system-specific microbial associations, infer potential ecological interactions, and compare the structural complexity and connectivity of microbial communities shaped by distinct agricultural practices. SparCC was considered more suitable for microbiome network analysis because it helps minimize compositionality bias and data sparsity, both of which are common in microbiome data, providing more reliable inferences on correlations in microbial abundance (Friedman and Alm, 2012). SparCC was computed at default settings using the SparCC Python codes available at https://github.com/dlegor/SparCC. Non-rarified ASV counts were utilized to calculate correlation coefficients and pseudo *p* values < 0.1. To generate a network, only significant correlations with associated pseudo *p* values of *p* < 0.1 were included using the igraph package (Csardi and Nepusz, 2006) in R. The network was transported and visualized in Cytoscape V.3.10.0 (Shannon et al., 2003) using the R package, Rcy3 (Gustavsen et al., 2019). Hub species in the networks of conventional and organic systems were identified as those falling within the top 10% of normalized betweenness centrality and degree of connectivity (*p* < 0.1), following the method outlined by (Agler et al., 2016).

### 2.7 Raman fingerprinting and phenotyping clustering of rhizomicrobiome associated with different plants

SCRS-based rhizomicrobiome phenotyping was performed for all the collected rhizosphere soil samples associated with different plants, with detailed protocol being demonstrated elsewhere (He et al., 2025). Briefly, soil samples were mixed with the detergent solution (3 mM sodium pyrophosphate, 0.5% Tween 20, 0.35% polyvinylpyrrolidone in PBS) and shaken for 30 min to detach the rhizo-microorganisms off the soil particles. The soil slurry was then placed on top of 80% Histodenz density-gradient solution (w/v) and subjected to centrifugation at 14,000 g under 4 °C for 1.5 h. The extracted and collected rhizo-microorganisms were then washed, diluted, and disaggregated by passing through 26-gauge needles repeatedly before being placed on top of the CaF_2_ Raman slide (Crystran Ltd., UK).

Raman instrumentation, setup, and data processing can be found in our previous publications (Majed and Gu, 2010; Li et al., 2018). A total of at least 100 Raman spectra with biological signature peaks at 1003 cm^−1^ and 1657 cm^−1^, corresponding to phenylalanine and amide I, respectively, were collected to represent the rhizomicrobial communities of each plant. OPUs, defined based on the similarity of Raman spectra to cluster and categorize microorganisms into different groups, were created through multivariate clustering as described previously (Li et al., 2018; He et al., 2025), except for the cutoff value adjusted to 0.44 according to Akaike information criterion (AIC) to account for the high diversity of the rhizomicrobiome. To evaluate the relationship between taxonomic and phenotypic alpha diversity metrics, we performed both linear and log-transformed linear regression analyses between ASV- and OPU-derived richness, Shannon, and evenness values. Ordinary least squares (OLS) regression models were fitted for each treatment using the statsmodels Python package (v0.14.1) (Seabold and Perktold, 2010). In the log-transformed models, both the independent and dependent variables were transformed using the natural logarithm of one plus the value (log1p) to accommodate zero values and improve numerical stability using Python Numpy (v2.2.4) (Harris et al., 2020). For each model, the coefficient of determination (R^2^) and corresponding *P* value were extracted to assess the strength and significance of the relationship. Heatmaps were generated using seaborn (v0.13.2) to visualize R^2^ values across treatments and diversity metrics, with statistical significance indicated by asterisks (*p* < 0.05 (^*^), *p* < 0.01 (^**^), *p* < 0.001 (^***^)). The dbRDA biplot was generated to assess significant Spearman correlations between different operational phylogenetic units (OPUs) and microbial abundance, utilizing the microeco package (v.1.8.1) (Liu et al., 2021). The direction and strength of each correlation of different OPUs with soil microbiomes were identified using the function *Envfit* (permutations = 999) in the package vegan (Oksanen et al., 2007). Notable correlations between the OPUs and individual microbial members were determined by *p* values < 0.1.

## 3 Results

### 3.1 Distinct rhizomicrobial taxonomic structures across different plants and production systems

A total of 1,709,785 ASVs were recovered from 110 samples, representing five replicates each of 10 conventionally and 12 organically grown plants, with an average of 15,544 ASVs per sample. A total of 4,151 bacterial species were discovered and belonged to 33 phyla, 267 families, and 460 genera. Taxonomic composition of the soil microbiome was analyzed across multiple hierarchical levels, comparing organic and conventional cultivation conditions across all plant species (Supplementary Figure 3). Most species were distributed across seven bacterial phyla, Acidobacteriota, Actinobacteriota, Bacteroidota, Chloroflexota, Gemmatimonadota, Proteobacteria, and Verrucomicrobiota, with others belonging to other phyla that make up less than 0.5% of the total abundance. At the family and genus levels, soil microbiomes displayed considerable diversity, with a broad spectrum of taxa shaping overall composition across different plant species and production systems. No singleton ASVs (i.e., ASVs observed only once across the dataset) were detected following quality control. Subsequent dual filtering, based on mean relative abundance >0.5% and prevalence in ≥80% of samples within each plant type group, yielded 80 core taxa, which were used for downstream analyses.

**Figure 1:**
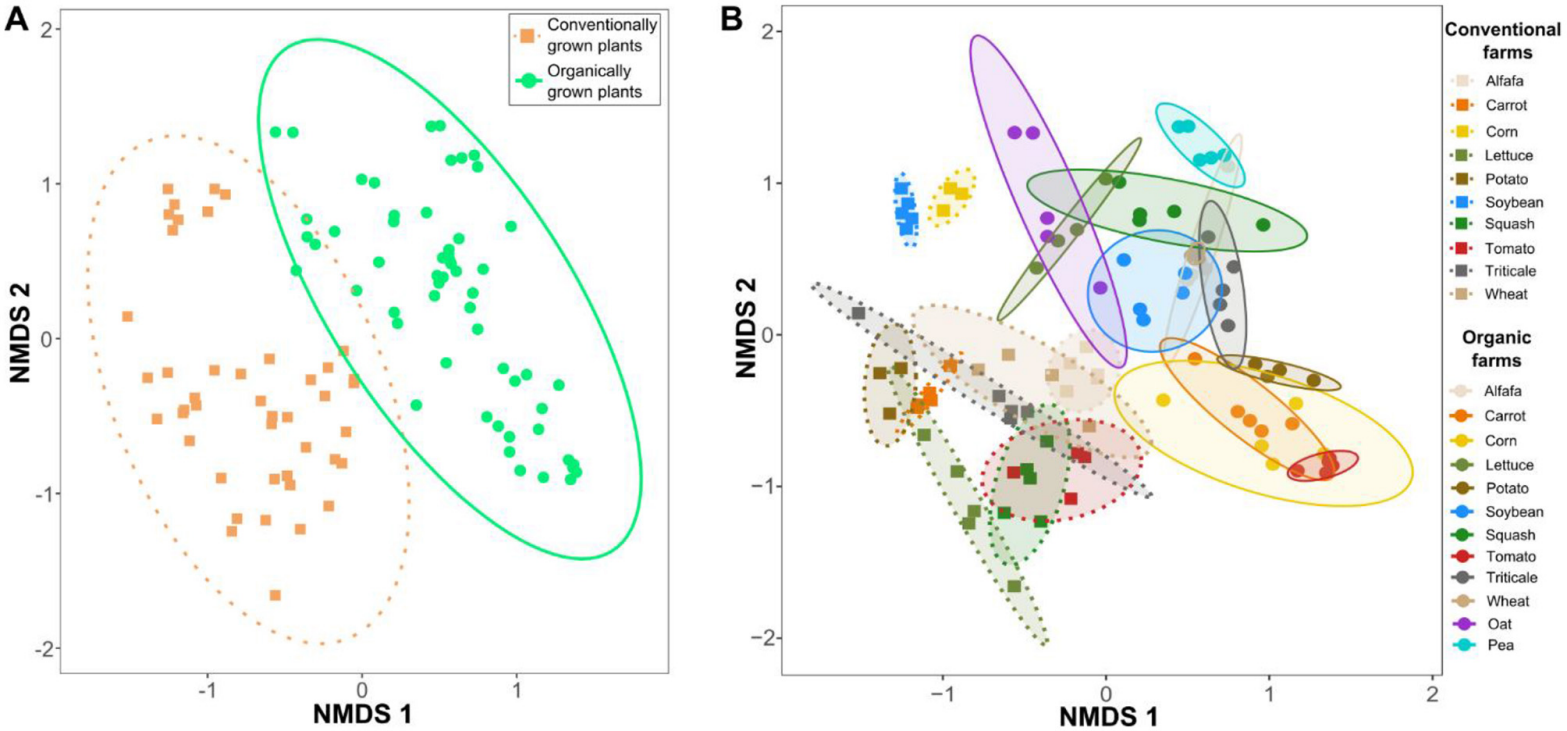
Non-metric multidimensional scaling (NMDS) ordinations based on Bray-Curtis dissimilarity illustrating variations in microbial community structures. **(A)** NMDS ordinations of all samples cultivated under conventional and organic agricultural systems. **(B)** Microbial community composition changes in response to interactions among plant species, production system, and site-level factors, indicating that ecological outcomes are shaped by both biotic and environmental contexts. Each group was enclosed within a 95% confidence ellipse based on standard deviation, illustrating within-group dispersion in ordination space. Ellipses were color-coded to correspond with their respective production categories.

Non-metric multidimensional scaling (NMDS) ordinations based on Bray-Curtis dissimilarity demonstrated clear differences in microbial community composition between conventional and organic systems, forming two distinct 95% confidence ellipses (Figure 1A). This suggests that farm production system (organic vs. conventional) played an important role in shaping microbial community structure, despite samples being collected from different locations and soil environments. Rhizosphere samples from plants grown under organic conditions showed greater compositional similarity to one another than to those from conventional systems. Furthermore, microbial community composition was further shaped by interactions between plant species and production systems, highlighting the co-influence of host plant identity and farming practices in structuring associated microbiomes (Figure 1B). A PERMANOVA analysis (model: Diversity ~ Production type × Plant species + Site latitude + Site longitude) yielded an R^2^ of 0.85 (*p* < 0.001), indicating that farming regime, plant species, and site-specific geographic variation collectively contributed to the observed diversity patterns. An ordination plot labeled by farm or geographic location has been generated to provide additional spatial context for the study (Supplementary Figure 4). Overall, these results emphasizes the dominance of farming practices in structuring soil microbial communities, with plant species acting as an additional but secondary driver of microbiome composition.

**Figure 2:**
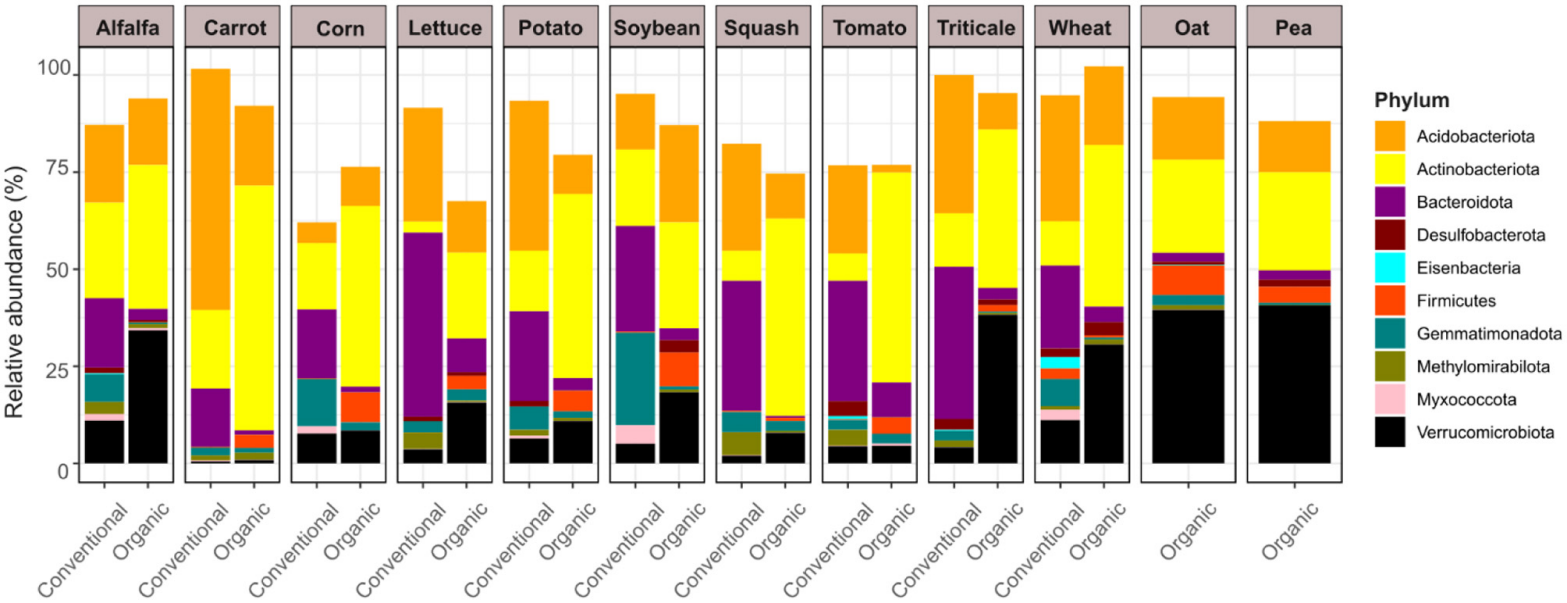
Differential abundance of microbial phyla across conventional and organic soils was assessed using ALDEx2. Statistical significance was determined via a generalized linear model incorporating multiple factors (Production type × Plant species interaction), with Benjamini-Hochberg (BH) adjusted *p* values < 0.05. Only Phyla exhibiting significant differences are shown, each color-coded according to its taxonomic classification.

Our differential abundance analysis using the R package ALDEx2 (Fernandes et al., 2013) modeled the interaction between farming system and plant species to account for sample heterogeneity across field conditions (Figure 2). This approach identified ten microbial phyla whose relative abundances were significantly associated with both plant identity and production style: Acidobacteriota, Actinobacteriota, Bacteroidota, Desulfobacterota, Eisenbacteria, Firmicutes, Gemmatimonadota, Methylomirabilota, Myxococcota, and Verrucomicrobiota. Despite variation in plant species and sampling sites, distinct phylum-level patterns emerged between conventional and organic systems. Acidobacteriota, Bacteroidota, and Gemmatimonadota were consistently more abundant in rhizosphere soils under conventional management, while Actinobacteriota, Firmicutes, and Verrucomicrobiota were enriched in organic rhizomicrobiomes. Specifically, Acidobacteriota bacteria were prevalent in conventionally grown carrot, lettuce, potato, squash, tomato, triticale, and wheat; Bacteroidota in alfalfa, carrot, corn, lettuce, potato, soybean, squash, tomato, triticale, and wheat; and Gemmatimonadota in alfalfa, corn, potato, soybean, and wheat. In contrast, Actinobacteriota were widely observed in organically grown alfalfa, carrot, corn, lettuce, potato, soybean, squash, tomato, triticale, and wheat; and Firmicutes in organic carrot, corn, lettuce, potato, soybean, and tomato. Verrucomicrobiota members were abundant in organic alfalfa, lettuce, potato, soybean, squash, triticale, and wheat, with notably high abundance also observed in the rhizospheres of organically grown oat and pea. A complete summary of ALDEx2 results is provided in Supplementary Table 1.

### 3.2 Key rhizomicrobial hub species revealed from network analysis in hyperdiverse rhizomicrobiome from conventional and organic agriculture

We constructed co-abundance networks within the rhizomicrobiomes of plants cultivated under conventional and organic agricultural systems to identify consistent microbial association patterns across production regimes. Then, we identified hub species within the microbial networks of conventional and organic systems, based on their placement in the top 10% for normalized betweenness centrality and degree of connectivity (*p* < 0.1) (Supplementary Figure 5), following the approach described by Agler et al. (2016). Betweenness centrality represents a taxon's function as a connector, facilitating interactions among microbial members, while degree of connectivity is related to its ecological importance based on extensive associations within the community (Agler et al., 2016; Delmas et al., 2019). Together, these metrics help pinpoint key microorganisms that play a structural and functional role in shaping microbial communities, referred to as hub species. These hub species function as important ecological components, contributing to the stability and sustainability of microbial community structure (Agler et al., 2016; Ahmed et al., 2021). Due to their high connectivity and central positioning within microbial networks, hub taxa play a pivotal role in ecosystem dynamics and stability, such that their loss can trigger widespread disruption across the entire microbiome (Agler et al., 2016; Delmas et al., 2019).

**Figure 3:**
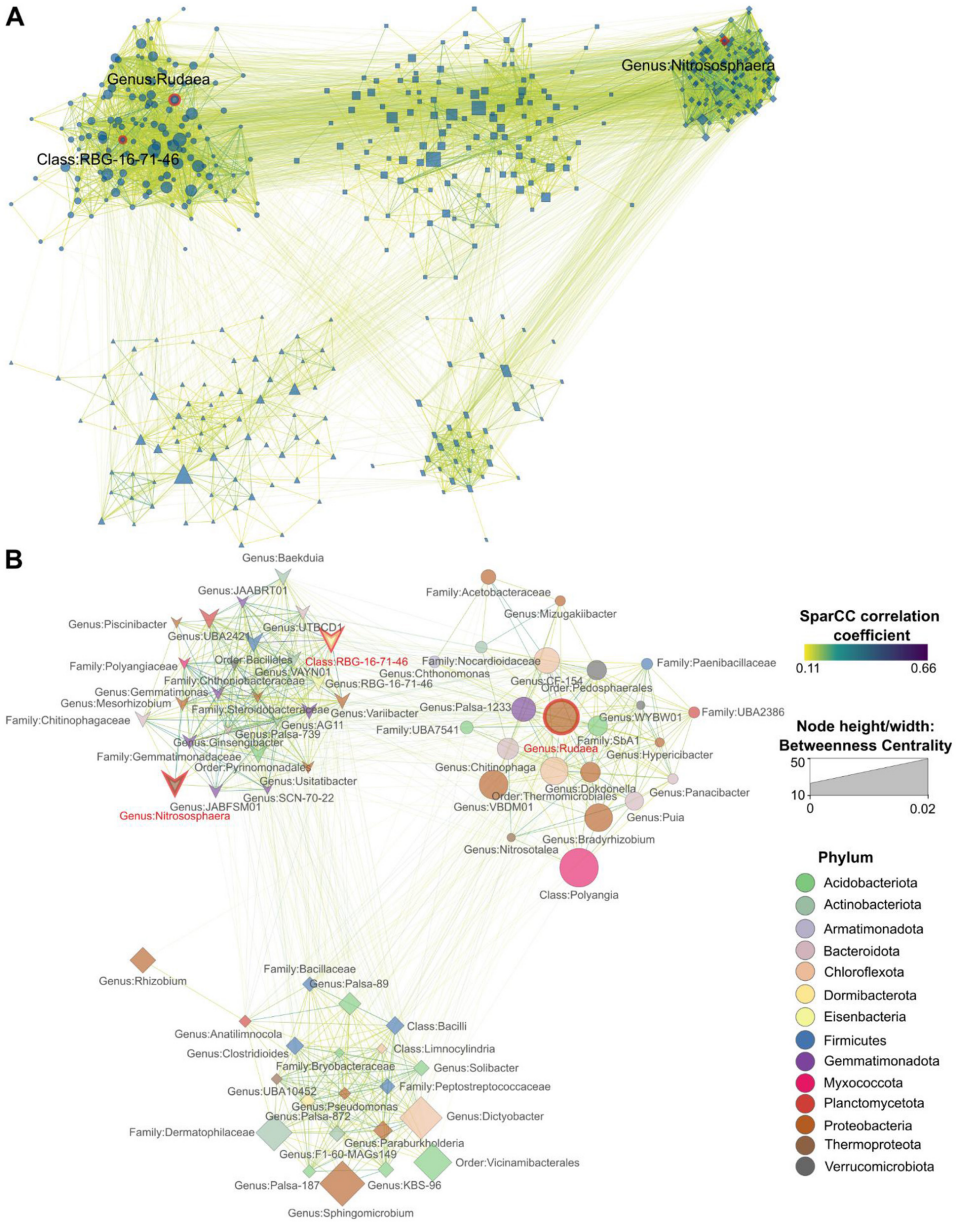
Microbiome SparCC co-abundance networks. **(A)** Co-abundance network of rhizomicrobiomes from plants cultivated under conventional agricultural conditions **(B)** The co-abundance network of the hub species in conventional farming systems. Hub species are annotated at their lowest taxonomic level and highlighted with red border lines and red text. Nodes represent individual taxa identified at their lowest taxonomic level, with node colors corresponding to their respective phyla. Node size reflects the betweenness centrality of each taxon, indicating its importance in microbial interactions. Edges connecting nodes are color-coded to represent their correlation strength. Only statistically significant correlations (pseudo *p* < 0.1, derived from bootstrapping with 100 repetitions) were included in each network. Clusters were generated using the Markov Clustering Algorithm (MCL), with each cluster's nodes depicted in different shapes to distinguish groupings within the network.

Using SparCC network analysis (Friedman and Alm, 2012), we constructed microbial co-abundance networks representing microbiomes from conventional and organic agriculture, referred to as the conventional network and organic network throughout the text. Figure 3A depicts the co-abundance network of rhizomicrobiomes from plants cultivated under conventional farming systems, comprising 483 soil microbial taxa engaged in 7,505 significant interactions. Within the conventional network, three hub microbial species were identified, including members of the genus *Rudaea*, genus *Nitrososphaera*, and class RBG-16-71-46. To further examine their role, we constructed a co-abundance network specifically linked to these hub species (hereafter referred to as hub network), which encompassed 769 co-abundance interactions with 66 microbial taxa (Figure 3B). Supplementary Table 2 provides details on co-abundance network interactions within the conventional hub network, including linked members, SparCC correlation coefficients (R), and *p* values. The strongest interactions in the hub network of rhizomicrobiomes in conventional systems were identified between members of the genus *Palsa-739* and the family Gemmatimonadaceae (*R* = 0.66), followed by the positive correlation between the class RBG-16-71-46 and the order Bacillales (*R* = 0.57); by the genus *Rudaea* and the genus *CF-154* (*R*= 0.56); by the family Steroidobacteraceae and the family Polyangiaceae (*R* = 0.55); by the genus *Mesorhizobium* and the genus *VAYN01* (*R* = 0.53); and by the genus *Rudaea* and the genus *Ginsengibacter* (R=0.52).

**Figure 4:**
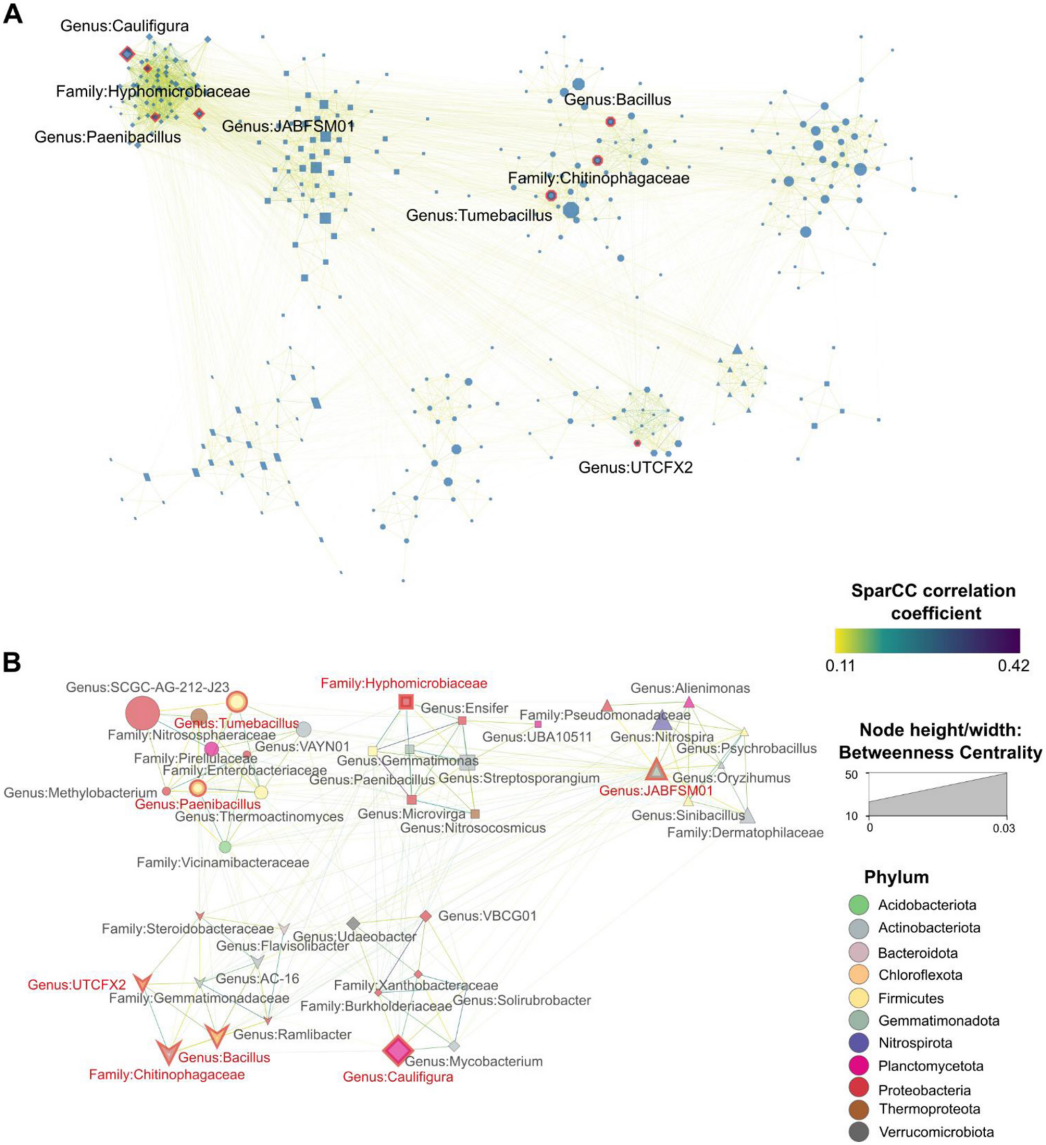
Microbiome SparCC co-abundance networks. **(A)** Co-abundance network of rhizomicrobiomes from plants cultivated under organic agricultural conditions. **(B)** The co-abundance network of the hub species in organic farming systems. Hub species are annotated at their lowest taxonomic level and highlighted with red border lines and red text. Nodes represent individual taxa identified at their lowest taxonomic level, with node colors corresponding to their respective phyla. Node size reflects the betweenness centrality of each taxon, indicating its importance in microbial interactions. Edges connecting nodes are color-coded to represent their correlation strength. Only statistically significant correlations (pseudo *p* < 0.1, derived from bootstrapping with 100 repetitions) were included in each network. Clusters were generated using the Markov Clustering Algorithm (MCL), with each cluster's nodes depicted in different shapes to distinguish groupings within the network.

We observed 348 microorganisms involved in 3,168 co-abundance interactions in the soil microbial communities of the plants grown under organic agriculture (Figure 4A). In the organic network, we discovered eight hub microbial species such as the genus *JABFSM01*, the genus *Tumebacillus*, the family Hyphomicrobiaceae, the genus *Caulifigura*, the genus *UTCFX2*, the genus *Bacillus*, the family Chitinophagaceae, and the genus *Paenibacillus*. In the hub network of microbiomes in organic agricultural systems, 41 microbial species were found to engage in 217 significant interactions with the eight hub species (Figure 4B). A detailed summary of all co-abundance interactions in the organic hub network, including linkage members, SparCC correlation coefficients (R), and *p* values, is provided in Supplementary Table 3. In the organic hub network, the strongest interactions were found between species associated with the genus *Microvirga* and the genus *Paenibacillus* (R = 0.42), followed by the genus *VBCG01* and the family Burkholderiaceae (R = 0.37); by the genus *Paenibacillus* and the genus *Ensifer* (R = 0.35); by the genus *Mycobacterium* and the genus *Solirubrobacter* (R = 0.31); by the genus *Paenibacillus* and the family Burkholderiaceae (R = 0.31); and the genus *Thermoactinomyces* and the genus *Paenibacillus* (R = 0.31).

### 3.3 Single-cell Raman fingerprints and phenotype clustering of rhizomicrobiome associated with different plants

**Figure 5:**
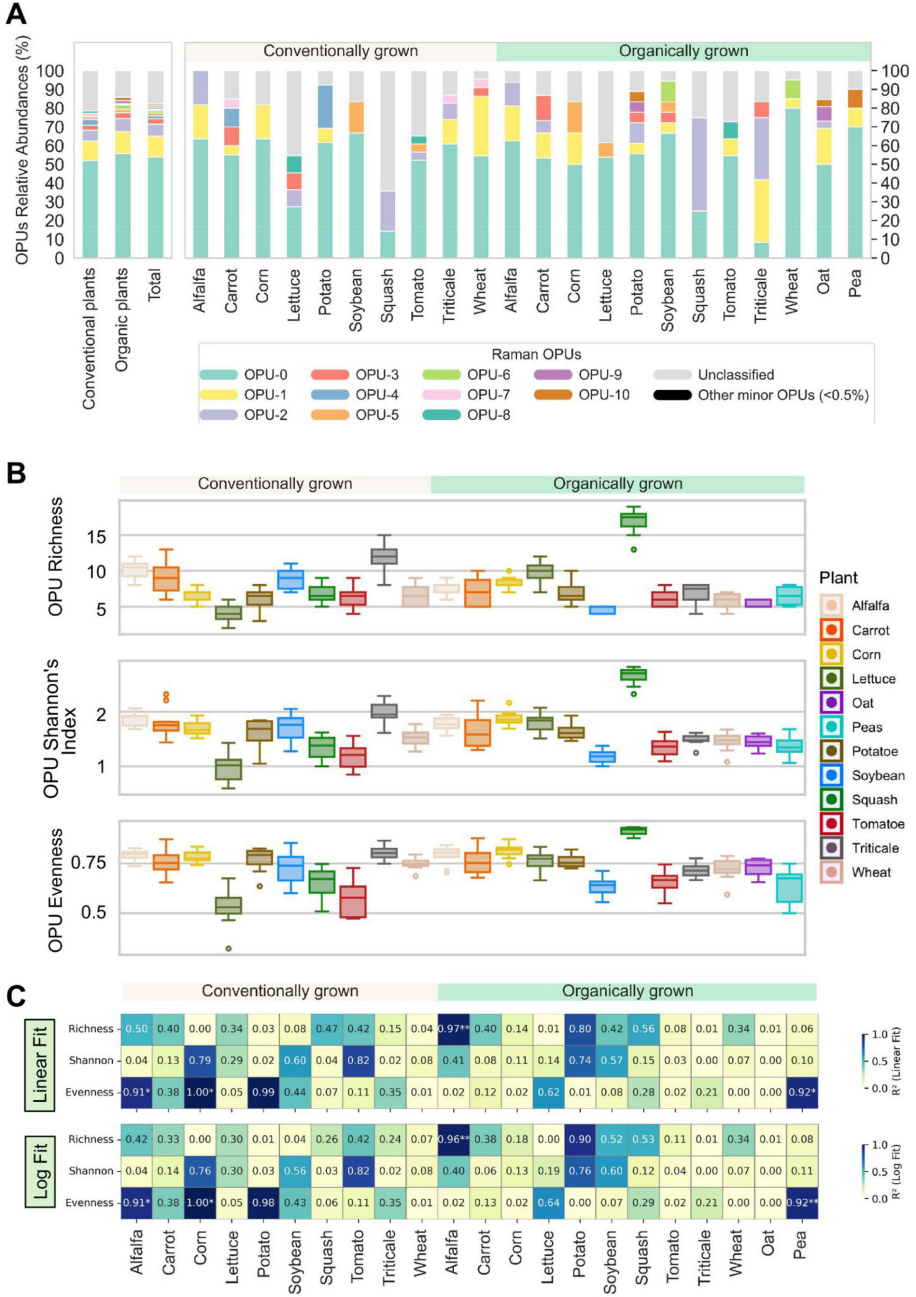
Characterization and comparison of Raman profiles of rhizomicrobiome. **(A)** Phenotypic profiling of the microbial community based on operational phenotypic units (OPUs) distribution among different crops surveyed. The left panel shows the OPUs distribution for conventional plants, for organic plants, and overall, while the right panel displays those for each individual crop. A total of 21 OPUs are identified and numerically labeled sequentially and only 12 OPUs with relative abundances over 0.5% are shown, while the other 9 minor OPUs are combined as “Other minor OPUs (<0.5%)”. Raman spectra that did not get classified into the 21 OPUs are combined as “Unclassified”. **(B)** Distribution of richness, Shannon's diversity index, and evenness of OPUs. **(C)** Heatmaps showing the coefficient of determination (R^2^) from linear and log-transformed linear regression models between ASV-based (taxonomic) and OPU-based (phenotypic) alpha diversity metrics across samples, revealing phenotype-genotype correlations. Each cell represents the R^2^ value for a given diversity metric (richness, Shannon, or evenness) within a treatment, fitted between ASV and OPU values. Significant differences were identified using both linear and log-transformed linear regression models. For all panels, significance is indicated by asterisks (*p < 0.05; **p < 0.01).

**Figure 6:**
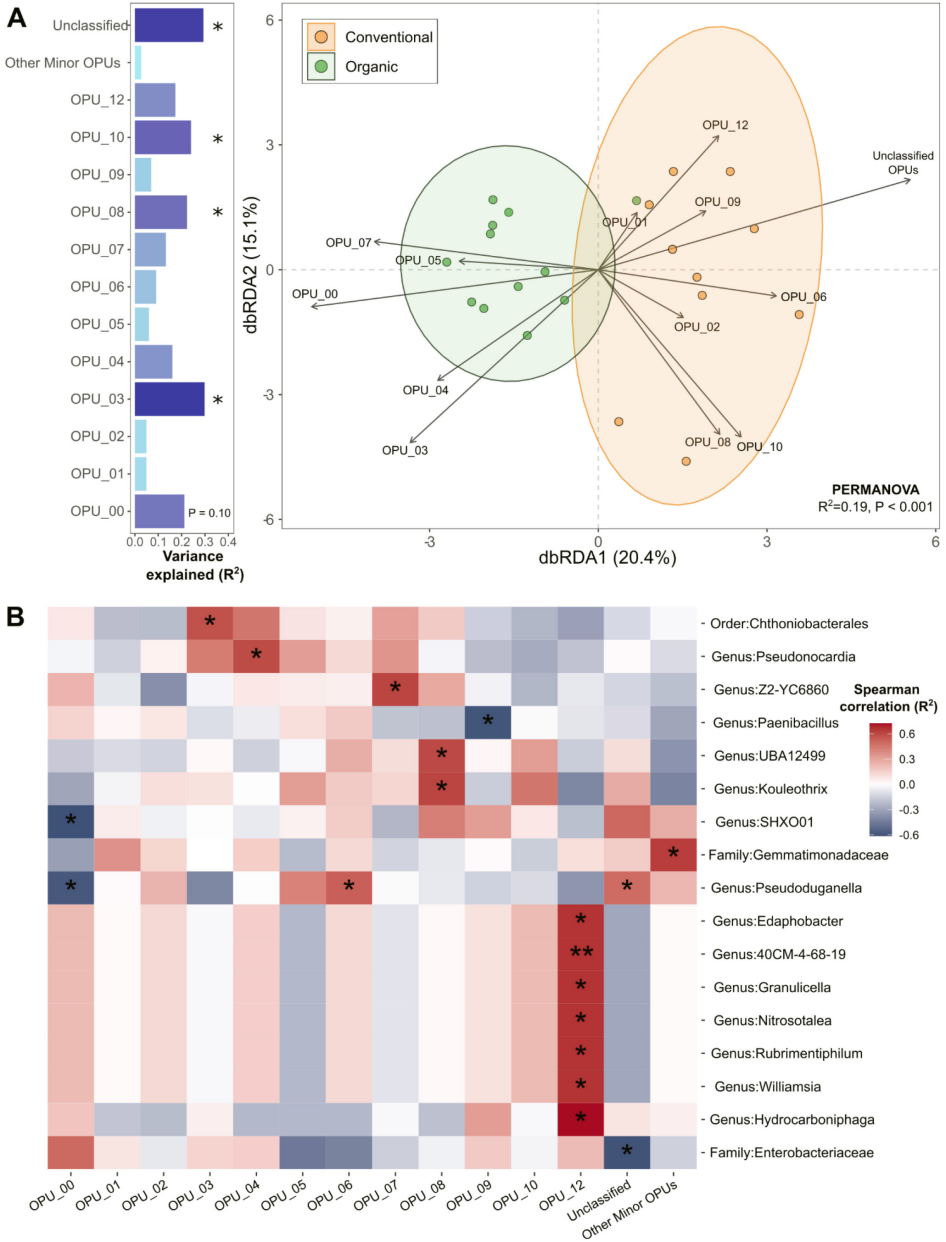
Microbiome variability and functional interactions in conventional and organic production systems. **(A)** Distance-based redundancy analysis (dbRDA) assessing the relationship between microbial community structures and OPU distributions, treating microbial phenotypes as environmental variables that drive differentiation between conventional and organic agricultural soils. The variance explained by each OPU was assessed using the EnvFit function in the vegan R package and depicted in horizontal bar graphs positioned to the left of the dbRDA plot. Asterisks indicate significant OPUs, showing functional groups that were differentially associated with microbial communities in conventional and organic systems at Benjamini–Hochberg (BH) adjusted *p* < 0.1. **(B)** Heatmap showing Spearman correlations between microbiome structure and OPU abundance. Significant microbial taxa were identified at the BH adjusted *p* < 0.1 (*p < 0.1; **p < 0.05).

We used SCRS to conduct single-cell phenotypic profiling of the rhizomicrobiome across various crops, generating 3,285 Raman spectra, each representing the metabolic fingerprint of an individual cell. While OTUs are defined by nucleotide sequence similarities, clustering microorganisms based on genetic markers (e.g., 16S rRNA gene for bacteria) to infer taxonomic relationships, OPUs are derived from phenotypic similarities observed in single-cell Raman spectra captured by SCRS. These spectral profiles reflect biochemical composition and metabolic activity, allowing for functional grouping independent of genetic sequence data. Hierarchical clustering grouped 66% of the collected spectra into 21 OPUs, while the remaining 34% were singletons with little similarity to any other spectra. The distinctive distribution of the identified OPUs of rhizomicrobime samples of all plants are shown in Figure 6A. Among the identified OPUs, OPU-0 and OPU-1 were both ubiquitous, occurring in all samples regardless of crop type or farm operation style, and abundant, collectively accounting for half of all acquired spectra (Figure 5A). The other OPUs were less abundant and not universally present among all plants. No significant differences (*P* > 0.05) were found in terms of the overall OPUs abundance distribution or the abundances of specific OPUs between conventional and organic plants.

Distinct Raman fingerprints could also be identified for each OPU, underscoring substantial variations in their cellular molecular compositions. Leveraging the Fisher rank score analysis and the correspondence between Raman shifts and certain biomolecular bonds or structures, especially in nucleic acids, protein, carbohydrates, and lipids (Cui et al., 2022), the metabolic and physiological implications of observed discrepancy in Raman spectra can be further elucidated (Supplementary Figure 6). For example, OPU-0 and OPU-1, though both prevalent, exhibited distinctly different Raman features. OPU-0 showed characteristic peaks around 1250 and 1450 cm^−1^, linked to CH_2_ structures in lipids (Cui et al., 2022), whereas OPU-1 was characterized by peaks at 1358 and 1574 cm^−1^, associated with amino acids and peptide bonds in proteins. OPU-8 phenotype was highly concentrated with the Raman spectra in ranges between 600-800 cm^−1^ related to peptides and proteins (tyrosine, amide IV, amid V, and tryptophan), nucleotides (guanine, adenine, uracil, thymine, cytosine), and cytochrome C (Cui et al., 2022). Among these, OPU-8 was most enriched at 687 cm^−1^ (23.1%), associated with trimethylsilyl (Si-CH) rocking vibrations (35), suggesting enhanced carbon uptake and metabolism in this phenotype (Bae et al., 2005).

The beta diversity of the microbial phenotypes associated with different plants was further elucidated by the SCRS-metabolites profiles as OPUs (Figure 5B). The richness, Shannon's index, and evenness were not significantly different between conventional and organic plants (*p* > 0.05). However, for specific plants, variations in the phenotypic structures between the soil microbial communities in conventional and organic farms showed that conventional lettuce and squash had lower richness, Shannon's index, and evenness than their organic counterparts (Supplementary Figure 7), although many other plants did not exhibit a notable difference between conventional and organic systems. To further assess the relationship between taxonomic and phenotypic diversity, we performed linear and log-log regression analyses between ASV- and OPU-derived diversity metrics (Figure 5C). Strong and significant couplings between taxonomic and phenotypic diversity indices were only observed in conventional alfalfa and corn as well as organic alfalfa and pea, but overall, the logarithmic model did not significantly or consistently outperform linear fitting (Supplementary Figure 8). This observation suggested that function-centric OPU-derived diversity metrics continued to increase with the increasing ASV diversity metrics without signs of plateauing, which might imply that microbial communities had not yet necessarily exhibit functional redundancy.

Rhizosphere microbiomes were associated with microbial functionality, as reflected by the distinct distribution patterns of abundant OPU communities across conventional and organic systems (Figure 6A). Figure 6A shows that 35.5% of the variation in microbial community structure was explained by distinct OPU members, emphasizing their influence in shaping the functional divergence of rhizosphere microbiomes under conventional and organic agricultural systems. We applied Distance-based redundancy analysis (dbRDA) to reveal how microbial community structures correlated with OPU distributions, uncovering key microbial phenotypic traits that distinguished conventional and organic agricultural systems, with statistical validation using PERMANOVA (R^2^ = 0.19, *p* < 0.001). This approach underscores the distinct microbial assemblage shifts driven by differing agricultural practices. Several OPUs such as OPU-1, OPU-3, OPU-8, OPU-10, and unclassified OPUs were significantly correlated with the variations of microbiomes (Envfit, permutation = 10,000, BH-adjusted *p* < 0.1). The direction of each OPU arrow in the dbRDA ordination space indicates the strength and direction of correlation between the OPU and the site points within the ordination configuration. Among the significant OPUs, conventional microbiomes were strongly associated with OPU-8, OPU-10, and unclassified OPUs, whereas organic microbiomes showed strong correlations with OPU-0 and OPU-3.

To further explore microbial-functional associations, we conducted Spearman correlation analysis, identifying significant microbial taxa linked to OPU abundance at the BH-adjusted *p* < 0.1 (Figure 6B). Among OPUs significantly associated with rhizomicrobiomes in organic systems, OPU-0 type was negatively correlated with species belonging to the genera *SHXO01* and *Pseudoduganella*, while OPU-3 showed a positive association with a species of the order Chthoniobacterales. For conventional microbiomes, OPU-8 displayed significant positive correlations with members of the genera *Kouleothrix* and *UBA12499*. Several OPUs which formed notable correlations with specific microbial genera included OPU-4 which was correlated positively with genus *Pseudonocardia* and OPU-6 which exhibited a positive relationship with genus *Pseudoduganella*. Similarly, OPU-7 was positively linked to genus *Z2-YC6860*, whereas OPU-9 showed a negative correlation with genus *Paenibacillus* OPU-12 was positively associated with multiple genera, including *Edaphobacter, 40CM-4-68-19, Granulicella, Nitrosotalea, Rubrimentiphilum, Williamsia*, and *Hydrocarboniphaga*.

## 4 Discussion

This study aimed to identify distinctive microbial phenotypes and community architectures emerging across heterogeneous field conditions and 22 horticultural plants, using a combined approach of 16S rRNA gene sequencing and SCRS to resolve microbiome differences between conventional and organic agricultural systems. Although soil type and site-specific variables are known to influence microbiome composition and function, a prior study found that soil chemistry explained only ~20% of the total variance, suggesting that nutritional status induced relatively minor shifts in community structure (Hartmann et al., 2015). While edaphic factors likely contributed to variation in our dataset and warrant further investigation, we prioritized capturing microbial traits that consistently emerged across heterogeneous conditions, highlighting system-specific signatures that distinguish conventional and organic agriculture. In attempts to control for confounding factors and account for environmental variability, we employed a statistical model incorporating “Production type × Plant species” interactions. This approach helped mitigate the influence of spatially confounded variables and disentangle biologically meaningful patterns from background environmental noise.

### 4.1 Contrasting soil microbial community structure in conventional and organic agroecosystems

The most notable finding of our study is that microbial community structures in conventionally grown plants closely resembled those from other conventionally cultivated plants, while those in organically grown plants were more similar to other organically cultivated plants (Figure 1A). These distinct clustering patterns emerged within their respective agricultural systems, despite variations in plant species and site conditions. This observation offers a broader ecological perspective, extending insights beyond single-plant analyses within a single site. Unlike many studies that focus on a single plant or a few plants at the same site, our research took a regional-scale approach, comparing 10 sets of horticultural plant species (alfalfa, carrot, corn, lettuce, potato, soybean, squash, tomato, triticale, and wheat) in conventional vs. organic systems, plus two additional crops (oat and pea) in organic systems. Despite soil and plant variability, we observed intriguing patterns, including an increase in Actinobacteriota, Firmicutes, and Verrucomicrobiota in organic plants, and a decrease in Acidobacteria, Bacteroidota, and Gemmatimonadota (Figure 2). These findings align with previous studies, although trends cannot be generalized among all plant species. For instance, other researchers reported the high abundances of Actinobacteriota and Firmicutes in the soils of organic cereal crops (Peltoniemi et al., 2021), finger millet managed through regenerative agriculture (Singh et al., 2023), and organically grown tomato that were suppressive against tomato corky root disease (Workneh and van Bruggen AH, 1994); Firmicutes in soils managed under organic systems for a long term (Hartmann et al., 2015; Pershina et al., 2015; Bonanomi et al., 2016); and Verrucomicrobiota in organic tea tree cultivation (Lin et al., 2021). These findings underscore the significant impact of agricultural management on soil microbial communities, underscoring distinct structural patterns between organic and conventional systems.

Copiotrophic bacteria, known for their ability to flourish in nutrient-rich conditions, showed clear distinctions between organic and conventional agricultural systems (Fierer et al., 2007). Copiotrophic microorganisms increased in both organic and conventional systems, with Firmicutes and Actinobacteriota more abundant in organic farms, while Bacteroidota showed higher levels in conventional systems, reflecting distinct microbial adaptations to agricultural conditions (Figure 2). Previous studies have also observed similar trends, showing that the increased abundance of Firmicutes in organic systems was associated with the use of composted manure and organic matter, which created a nutrient-rich environment that supports their growth (Hamm et al., 2016; Armalyte et al., 2019). Similarly, many species of the Actinobacteriota are known to prosper in environments with high soil organic matter and nutrient availability (Workneh and van Bruggen AH, 1994; Bhatti et al., 2017; Hozzein et al., 2019). A meta-analysis of 56 peer-reviewed studies, encompassing 149 pairwise comparisons across diverse climatic zones and experimental durations (ranging from 3 to over 100 years), reported that organic systems had 32% to 84% higher microbial biomass carbon, microbial biomass nitrogen, total phospholipid fatty acids, and enzymatic activities (dehydrogenase, urease, and protease) compared to conventional systems (Lori et al., 2017). These findings indicate that organic farming enhances nutrient availability, which may contribute to the proliferation of copiotrophic microorganisms such as Firmicutes and Actinobacteriota. Conversely, Bacteroidota were more prevalent in conventional agriculture, as observed in previous studies (Pershina et al., 2015; Chen et al., 2023), likely due to the nutrient-rich conditions created by synthetic fertilizers, which support their growth. In this context, both conventional and organic agriculture provided nutrient-rich environments, as reflected in the abundance of copiotrophic bacteria. However, variations in microbial composition may result from distinct nutrient preferences, with Firmicutes and Actinobacteria thriving on organic matter-derived nutrients, while Bacteroidota may preferentially utilize nutrients directly from fertilizers. Collectively, both conventional and organic systems supported copiotrophic bacterial growth, but variations in nutrient sources and farming inputs likely shaped microbial community composition and adaptation patterns differently.

Our study revealed a high abundance of Verrucomicrobiota in the rhizosphere of organic agricultural plants compared to conventional ones (Figure 2). These enigmatic microorganisms are well-adapted to nutrient-poor environments, efficiently utilizing available resources in ways that enable their survival where many other microbes struggle (Shade et al., 2012; Navarrete et al., 2015; Shen et al., 2017). Particularly, their ability to process complex organic compounds makes them to play a crucial role in soil carbon cycling, contributing to nutrient turnover and long-term soil health (Fierer et al., 2013; Cai et al., 2024; Tan et al., 2025). The low-input nature of organic agriculture, with its gradual nutrient release, may create ideal conditions for these bacteria. Studies suggest that organic soils treated with low-rate organic fertilizers (0.7 LU per hectare) tended to maintain lower carbon and nitrogen levels, favoring the growth of oligotrophic microorganisms such as Verrucomicrobiota (Lori et al., 2023). The gradual nutrient release in organic farming regulated by composted manure, plant residues, and organic fertilizers, may generate localized nutrient-rich zones that promote copiotrophic bacterial growth while simultaneously sustaining low-nutrient pockets that favor oligotrophs. This dynamic nutrient distribution enables diverse microorganisms to thrive under resource-limited conditions, aligning with our findings.

### 4.2 Co-abundance patterns reflecting ecosystem characteristics and farming practices

Co-abundance networks analyze consistent microbial community patterns by detecting taxa that reliably co-occur across samples, uncovering ecological relationships such as mutualistic interactions or shared environmental dependencies (Hartmann et al., 2015). Only three hub species were identified in the conventional network, suggesting that hub taxa were less resilient under conventional practices, shifting readily in response to external pressures (Figure 3). Factors such as agrochemical applications and tillage likely imposed constraints, influencing which microbial taxa could establish central roles within the network (Kraut-Cohen et al., 2020). The presence of eight hub species in the organic network—more than twice the number found in conventional systems (3)—suggests that organic farming, devoid of agrochemicals and extensive tillage, may reduce selective pressure, allowing a broader range of hub taxa to be shared among organically grown plants rather than favoring a few dominant species (Figure 4). These shared ecological characteristics among organically grown plants may contribute to the stability of hub networks, ensuring resilience even amid environmental fluctuation (Philippot et al., 2021).

In conventional agricultural systems, our study identified numerous beneficial microorganisms within their hub network, despite the widespread use of agrochemicals such as pesticides and synthetic fertilizers (Figure 3). These include key microbial groups involved in nitrogen fixation (e.g., *Rhizobia, Bradyrhizobia, Mesorhizobium*, and *Paraburkholderia*) (Laranjo et al., 2014; Lindström and Mousavi, 2020; Bellés-Sancho et al., 2023), nitrogen cycling (e.g., *Nitrososphaera*) (Tourna et al., 2011; Reyes et al., 2020), and plant growth promotion (e.g., *Pseudomonas*) (Preston, 2004). It is surprising that more free-living nitrogen fixers were co-abundantly integrated across diverse plant rhizospheres in fields managed with synthetic fertilizers than within the organic hub network. Previously, inorganic nitrogen fertilizers strongly suppress free-living nitrogen fixation, since they favor the energetically cheaper inorganic nitrogen from fertilizers over atmospheric N_2_ fixation (Dynarski and Houlton, 2018). This finding contrasts with our current observation that nitrogen-fixing bacteria were abundant and centrally positioned within the microbial hub network of conventionally managed systems. However, a comparative study between chemical and organic fertilizers has shown that copiotrophic nitrogen fixers, such as *Bradyrhizobium* and *Burkholderia*, can exploit inorganic fertilizer-derived nutrients to fuel their vegetative growth rather than engaging in the energetically costly process of nitrogen fixation (Fan et al., 2019). In contrast, organic amendments (e.g. cow and pig manures) exerted milder suppressive effects on nitrogen fixation, coinciding with a higher prevalence of oligotrophic nitrogen fixers such as *Geobacter* and *Anaeromyxobacter* in organically managed soils. Based on these results, we conclude that *Rhizobia, Bradyrhizobium, Mesorhizobium*, and *Paraburkholderia* were likely associated with copiotrophic nitrogen-fixing strategies, thriving in nutrient-rich soils supplemented with fertilizers where they prioritize growth over nitrogen fixation.

Observations from the conventional hub network revealed that xenobiotic-degrading microorganisms such as *Pseudomonas* and *Mesorhizobium* (Wang et al., 2022) were consistently present across the rhizospheres of multiple plant species (Figure 3). Xenobiotics are chemical substances not naturally present in ecosystem. In agricultural settings, many of them are derived from synthetic pesticides, herbicides, fungicides, and insecticides such as atrazine, a widely applied herbicide, and hexachlorobenzene, a fungicide known for its high bioaccumulation potential and detection in soil, water, and even human milk (Wang et al., 2022). Several strains of *Pseudomonas*, including *Pseudomonas putida* MAS-1 being the most effective, have been shown to degrade chlorpyrifos, an organophosphorus pesticide widely used in agriculture and linked to adverse effects such as impaired seedling development, fruit deformities, and disrupted cellular division (Gilani et al., 2016). *Pseudomonas* is a metabolically diverse genus equipped with an extensive array of catabolic pathways and enzymes that facilitate the breakdown of various pesticide compounds. The presence of xenobiotic-degrading microorganisms in the conventional hub network suggests that the frequent input of agrochemicals possibly led to the enrichment of xenobiotic microorganisms that can degrade and mineralize these chemicals (Gianfreda and Rao, 2008). Despite the extensive application of agrochemicals in conventional systems, beneficial microorganisms, such as nitrogen fixers, nutrient cyclers, PGPR, and xenobiotic degraders, continued to play a crucial role in microbial networks.

In the organic network, we found several hub species, including Bacilli class members such as the genera *Tumebacillus, Bacillus, Paenibacillus*, and *Brevibacillus*, which are known as PGPR for their beneficial traits such as nutrient mineralization and disease control (Govindasamy et al., 2010; Pérez-García et al., 2011; Ray et al., 2020) (Figure 4). Our findings align with multiple prior studies that have reported high abundances of these Bacilli species in organic agricultural soils (Hartmann et al., 2015; Chaudhari et al., 2020; Peltoniemi et al., 2021; Sangiorgio et al., 2021), further supporting the strong association between organic amendments and Bacilli community dynamics. Additionally, we discovered several microorganisms included in the organic hub network, capable of carrying antibiotic-resistant genes (ARGs), including species belonging to the genus *Mycobacterium*, the family Enterobacteriaceae, the family Vicinamibacteraceae, the family Burkholderiaceae, and the family Steroidobacteraceae (Liu et al., 2019; Sangiorgio et al., 2021; Wang et al., 2024). The presence of ARG-associated microorganisms shared among the rhizospheres of organically grown plants may be linked to the frequent application of animal manures, a practice known to contribute to the spread of ARGs (Lima et al., 2020). This practice may accelerate the dissemination of ARGs originating from animal husbandry through the application of animal manures in organic agricultural systems, thereby contributing to the emergence and persistence of antibiotic-resistant bacteria. The presence and distribution of these microbial hosts in organic agricultural soils raise ongoing concerns regarding their potential impact on microbial ecosystems and resistance dissemination. Therefore, while organic farming enhances soil health through enriched PGPR activity, it may concurrently elevate the risk of ARG via manure-based inputs, highlighting a critical trade-off between ecological sustainability and microbial safety.

### 4.3 Linking microbial phenotypes to farming practices using a SCRS-based approach to soil microbiomes

Comparative analysis of organic and conventional agricultural practices using SCRS revealed distinct microbial phenotypes characteristic of each system. Organic agricultural soils were enriched with the phenotypes of lipid-accumulating microorganisms (OPU-0) which are possibly efficient in lipid accumulation owing to enhancing conversion of substrate to triacylglycerols or polyhydroxyalkanoates (PHAs), fatty acid synthesis, and intracellular lipid storage (Garay et al., 2014) (Figure 6A). This finding is consistent with our observations of increased growth of Actinomycetota and the actinomycete genus *Mycobacterium* (a key member of the organic hub network) in organically managed soils (Figures 2, 4). Actinomycete species possess the capability for *de novo* fatty acid biosynthesis from acetyl-CoA and can store substantial amounts of triacylglycerols, sterol esters, or long-chain lipid mycolic acids (C_60 − 90_) within their cells (Wältermann et al., 2005; Kalscheuer et al., 2007; Gago et al., 2011; Garay et al., 2014). The ability to store intracellular lipids benefits the survival of these microorganisms during starvation periods, as lipids serve as an important energy substrate, aiding in cell growth, division, and stress resilience under nutrient-limiting conditions (Garay et al., 2014). For instance, in *Legionella pneumophila*, the RNA-binding regulator CsrA is highly active during nutrient-rich, replicative growth, promoting amino acid catabolism (e.g., serine via the TCA cycle) and Entner–Doudoroff–mediated glucose degradation (Häuslein et al., 2017). Upon amino acid starvation, CsrA activity is relieved, enabling the upregulation of glycerol and fatty acid metabolism including polyhydroxybutyrate biosynthesis, which serves as a carbon and energy reserve. This metabolic shift explains how microorganisms, when faced with nutrient stress, mobilize dense, hydrophobic carbon reserves (e.g., lipids) into crucial survival fuel, powering stress-response pathways and sustaining viability under starvation. Thus, the prevalence of lipid-accumulating phenotypes under organic agricultural systems likely reflects microbial survival strategies in environments where nutrient availability fluctuates due to the gradual release of nutrients from organic materials commonly applied in these systems.

While the mechanisms driving lipid accumulation in microorganisms within organic systems remain unclear in the current study, it is plausible that the use of organic fertilizers (e.g. composted manure or plant-derived amendments) creates conditions that favor the proliferation of lipid-storing microbial taxa. A recent study comparing maize soils amended with organic fertilizers derived from cow manure to those treated with chemical fertilizers reported elevated levels of lipids and lipid-like metabolites, such as lithocholic acid, alongside increased concentrations of carbohydrates and oxygenated organic molecules in the organically managed soils (Wei et al., 2025). Organic materials often contain diverse and complex substrates, including lipid-rich residues such as plant-derived oils, waxes, sterols, and fatty acids originating from microbial biomass and decomposed organic matter (Ghouila et al., 2019). Organic fertilizers may supply lipid-rich substrates that stimulate lipid-utilizing microorganisms, such as members of Actinomycetota, thereby promoting the activation of lipid-metabolic pathways within soil microbial communities.

Conversely, conventional soils showed higher associations with a carbon-rich phenotype (OPU-8) (Figure 6A), such as those involving the genus *Kouleothrix* and the genus UBA12499, which belongs to the phylum Methylomirabilota (Figure 6B). These microorganisms are known to be efficient in carbon metabolism and storage, with *Kouleothrix* playing a significant role in carbon polymer hydrolysis and fermentation (Chen et al., 2024), and Methylomirabilota members utilizing volatile fatty acids for methanogenesis (Zhu et al., 2022). These metabolic processes supported microbial activity in conventional systems, enabling them to efficiently convert available carbon into energy, thereby sustaining immediate growth and functionality. Such phenotypes differed from those in organic systems, which favored lipid-accumulating microorganisms adapted for long-term energy conservation, whereas conventional systems were characterized by active metabolism and energy conversion. The frequent inputs of synthetic fertilizers and agrochemicals in conventional agricultural practices may ensure a steady supply of readily accessible nutrients, driving microbial activity toward faster energy extraction and storage.

We surveyed soil microbiomes in the rhizosphere of 10 conventionally and 12 organically cultivated crop species across conventional and organic farming systems, revealing distinct differences in microbial composition, network architecture, and phenotypic traits, despite variability in soil and plant types. Additionally, our integration of SCRS with microbiome analysis offers a novel approach for linking microbial phenotypes with genotypes, advancing the ability to characterize functional diversity across conventional and organic systems. By uncovering distinct co-abundance features—such as Bacilli hub taxa and ARG-carrying microorganisms in organic networks, and xenobiotic degraders alongside free-living nitrogen fixers in conventional networks—as well as system-specific microbial adaptations (e.g., lipid accumulation in organic soils and carbon-cycling phenotypes in conventional soils), our study lays the groundwork for future investigations into biochemical and molecular mechanisms underlying plant-microbe interactions. Although our study does not provide direct causal evidence between agricultural practices and individual microbial phenotypes, it underscores the need for further research into biochemical and molecular mechanisms shaping plant-microbe interactions, as well as additional studies on how agricultural practices influence these processes.

## Data Availability

The datasets presented in this study can be found in online repositories. The names of the repository/repositories and accession number(s) can be found here: https://www.ncbi.nlm.nih.gov/, BioProject: accession number PRJNA1263029 and PRJNA1263029
