# Supplementary material for: Geno-pheno characterization of crop rhizospheres: an integrated Raman spectroscopy and microbiome approach in conventional and organic agriculture
Source: Front Microbiol. 2025 Nov 28;16:1721013. doi: 10.3389/fmicb.2025.1721013 (PMC12698637; doi:10.3389/fmicb.2025.1721013)
Supplement: Supplementary file 1 [file Table_1.docx]

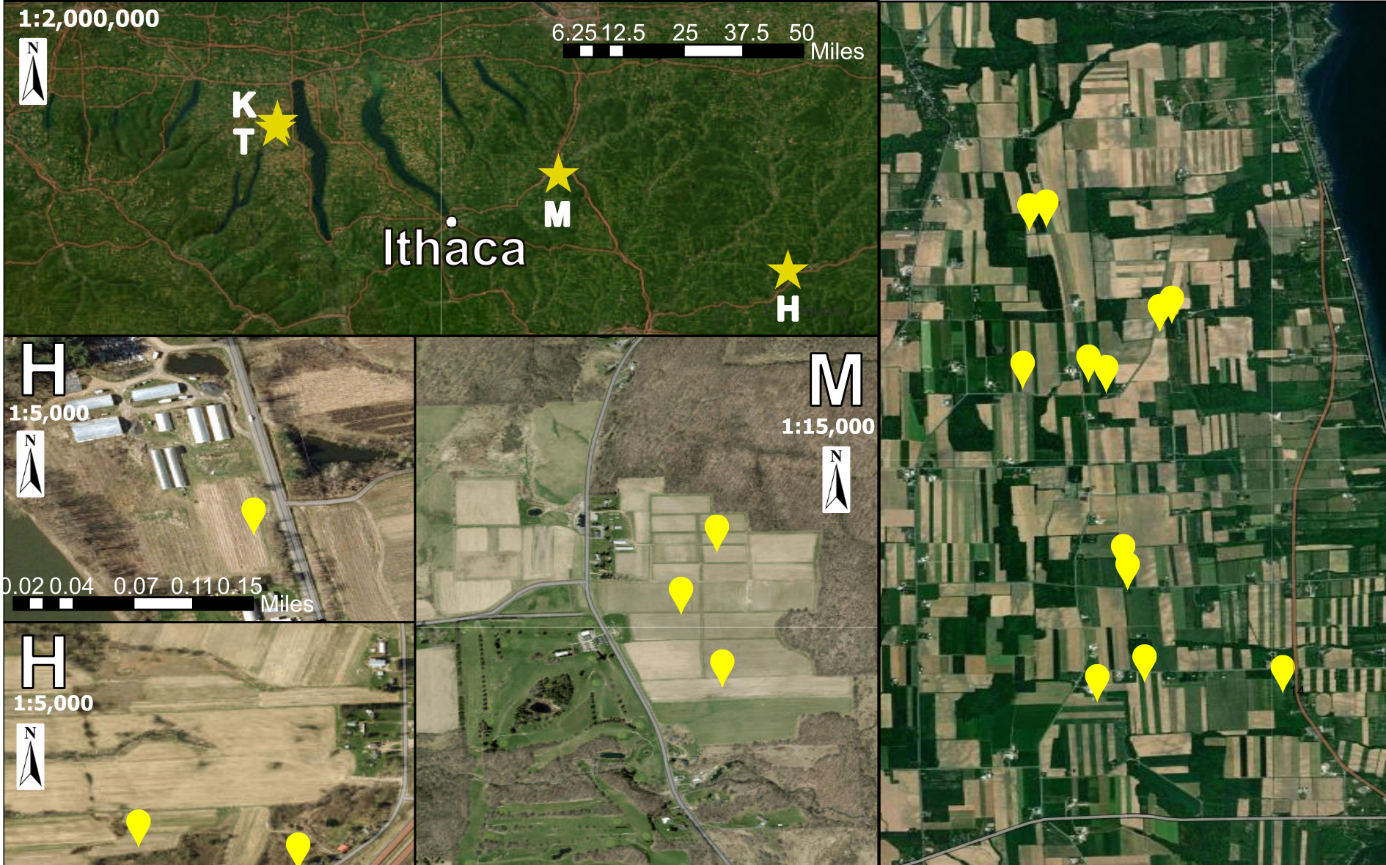


**Supplementary Figure 1.** Farm locations of organic and conventional systems. Abbreviations of sites: M for Mainstreet farms, K for Lakeview Organic Grain LLC, T for the conventional farm operated by Titus Zimmerman, and H for Hellers Farm CSA. The map is created by ArcGIS Pro 3.4 (Esri Inc., US) using the Imagery Hybrid map from ArcGIS Online.


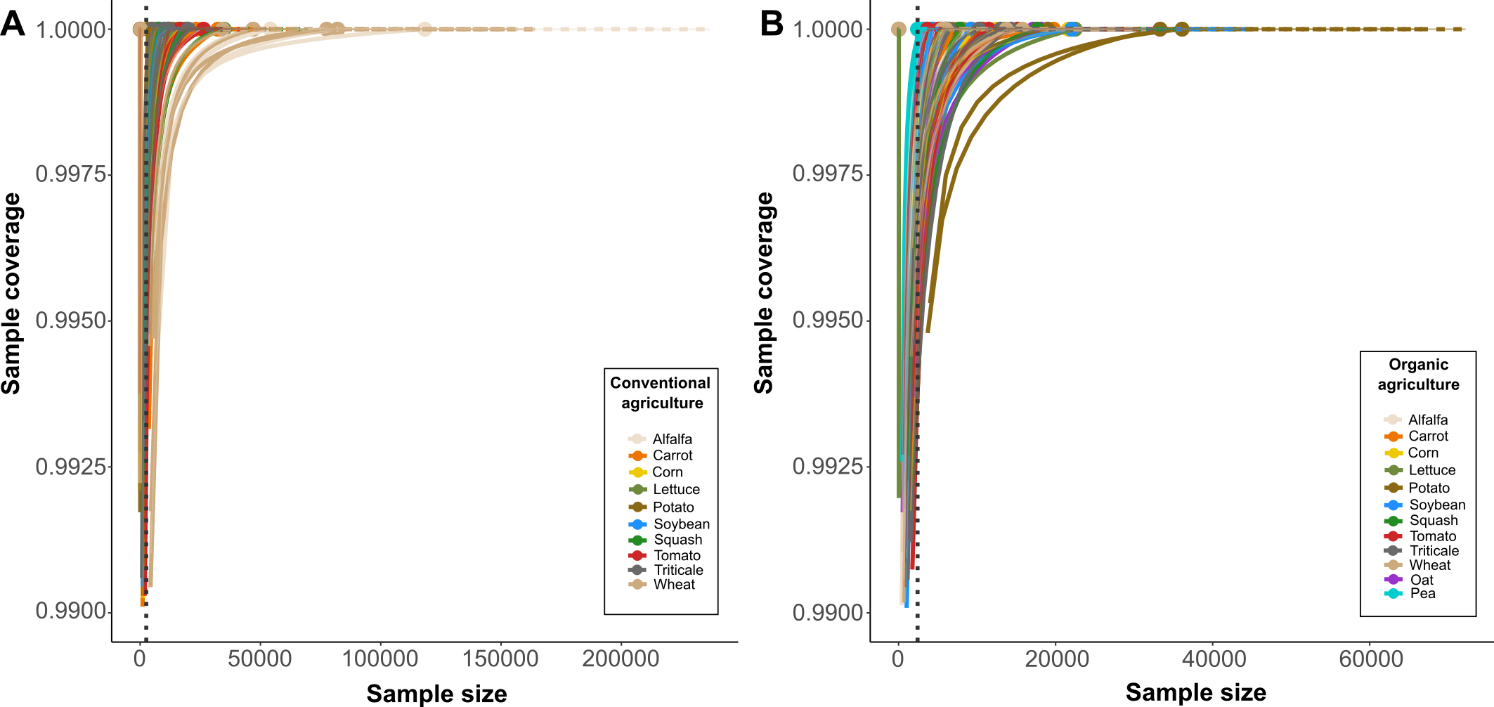


**Supplementary Figure 2.** Assessment of sample coverage after coverage-based rarefaction and extrapolation (R/E) for beta diversity, using Hill number (q=1, Shannon diversity). (A) The R/E curve of rhizomicrobiomes of conventionally grown plants. (B) The R/E curve of rhizomicrobiomes of organically grown plants. A coverage value above 0.99 on the y-axis indicates that the sequencing effort has successfully captured nearly all detectable species (> 99%) within each sample. Solid lines represent coverage-based rarefaction for each plant, while dotted lines illustrate extrapolated diversity, estimating Shannon diversity beyond the observed sequencing depth. The black dotted line indicates a rarefaction level of 2422 sequences.


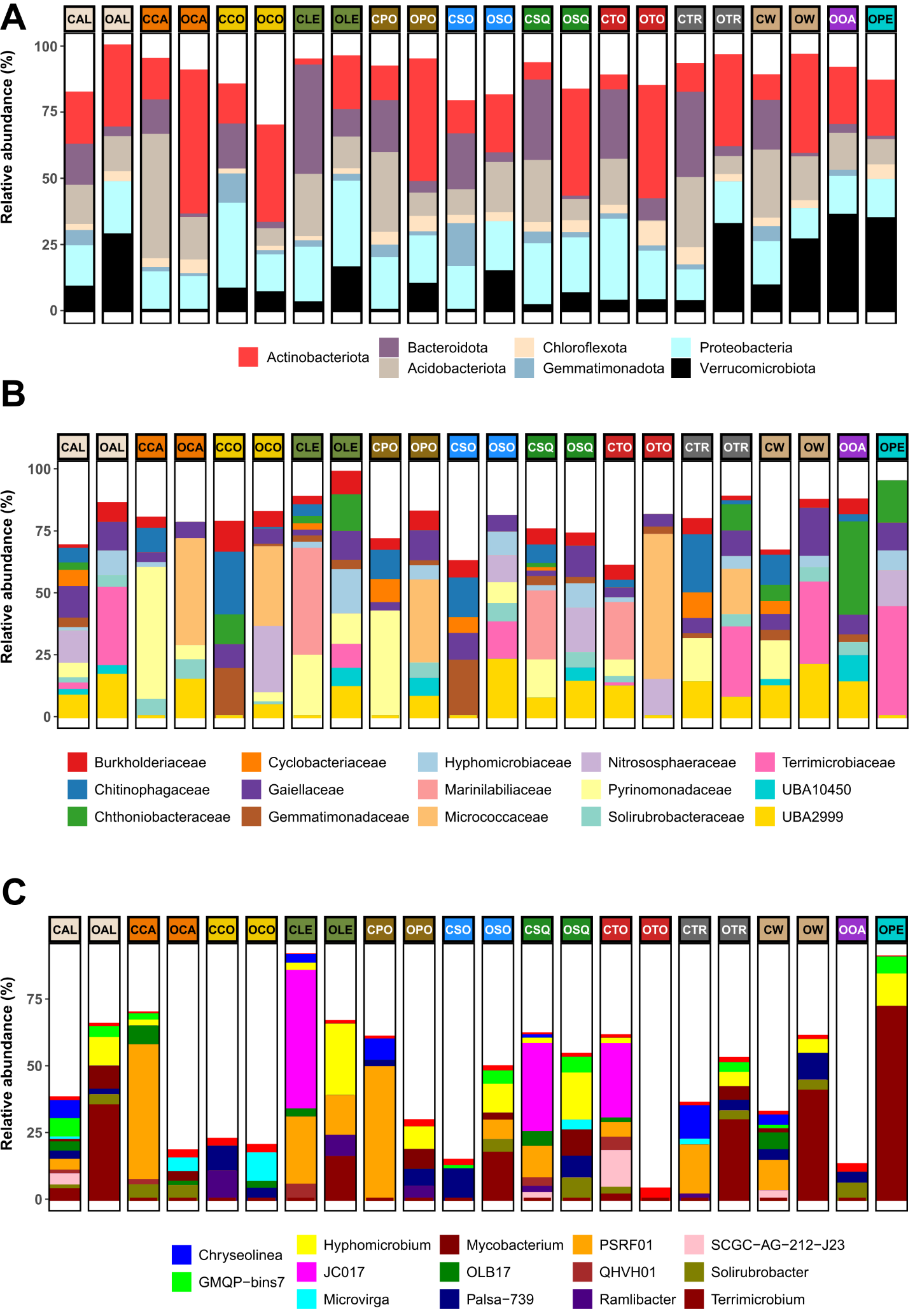


**Supplementary Figure 3.** Bar plots showing the average relative abundance of soil microbial taxa exceeding 0.5% of total abundance. Panel (A) presents distributions at the phylum level, while panels (B) and (C) display the top 15 most abundant taxa at the family and genus levels, respectively. Each plant is presented alongside its conventional and organic counterparts, except for oat and pea, which are displayed separately due to the absence of conventional counterparts. Abbreviations include CAL and OAL (conventional and organic alfalfa); CCA and OCA (conventional and organic carrot); CCO and OCO (conventional and organic corn); CLE and OLE (conventional and organic lettuce); CPO and OPO (conventional and organic potato); CSO and OSO (conventional and organic soybean); CSQ and OSQ (conventional and organic squash); CTO and OTO (conventional and organic tomato); CTR and OTR (conventional and organic triticale); CW and OW (conventional and organic wheat); OOA (organic oat); and OPE (organic pea).


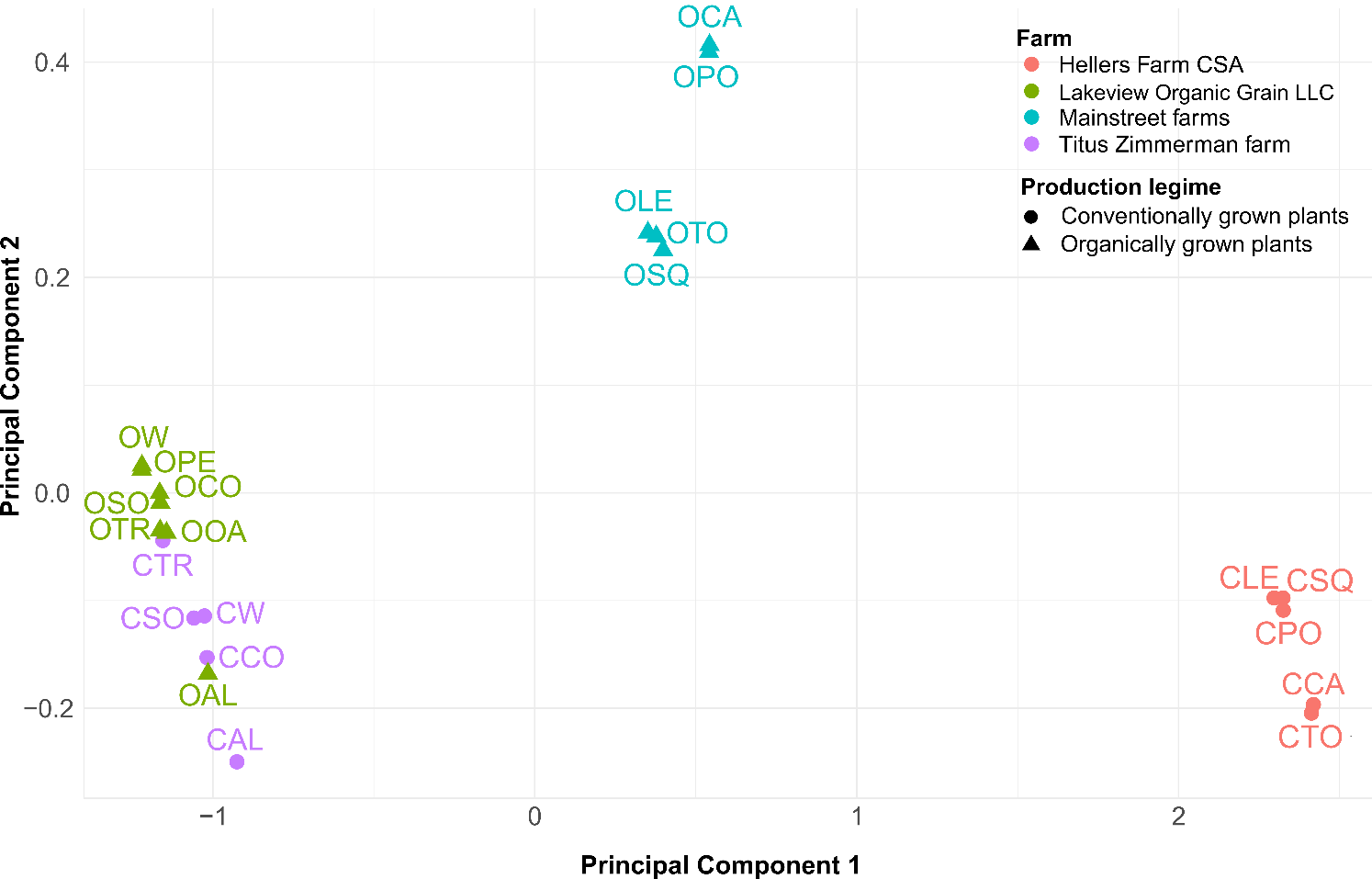


**Supplementary Figure 4.** Principal component analysis (PCA) of farm geographic distribution based on sampling site latitude and longitude. Each point represents a sample, colored by farm and shaped according to plant production style (conventional vs. organic). The ordination illustrates spatial variation in sampling sites across locations and management systems. Abbreviations include CAL and OAL (conventional and organic alfalfa); CCA and OCA (conventional and organic carrot); CCO and OCO (conventional and organic corn); CLE and OLE (conventional and organic lettuce); CPO and OPO (conventional and organic potato); CSO and OSO (conventional and organic soybean); CSQ and OSQ (conventional and organic squash); CTO and OTO (conventional and organic tomato); CTR and OTR (conventional and organic triticale); CW and OW (conventional and organic wheat); OOA (organic oat); and OPE (organic pea).


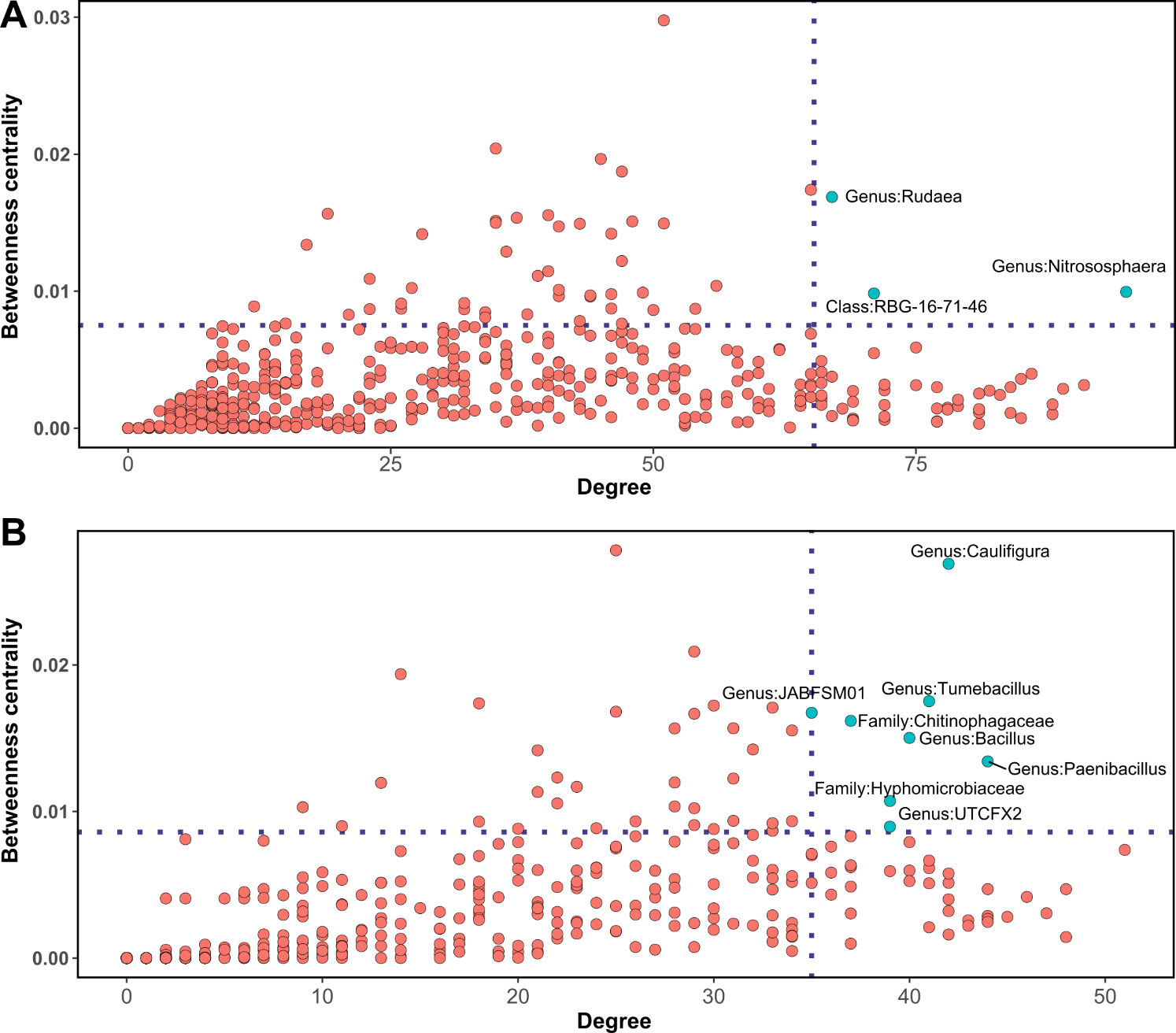


**Supplementary Figure 5.** Hub species in soil microbial networks identified by the highest betweenness centrality and node degree (*p* < 0.1). (A) Hub species of soil microbial communities of the plants grown under conventional agricultural systems. (B) Hub species of the soil microbial communities of the plants grown under organic agricultural systems. Hub members are represented in blue, while non-hub species are shown in red.

ss
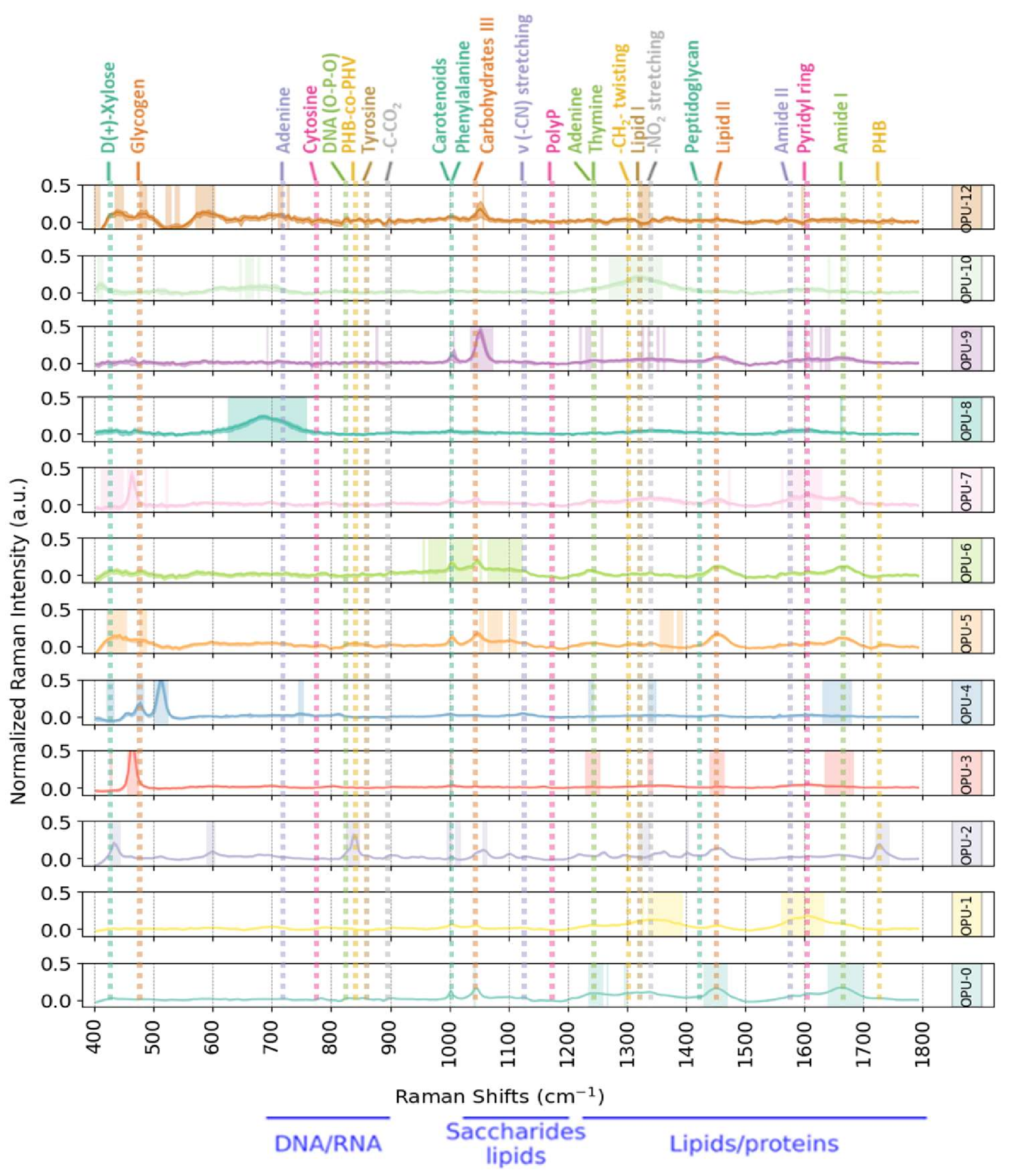


**Supplementary Figure 6.** The averaged Raman spectra of dominant OPUs. Highest 10% Fisher rank scores of variances between different OPUs over among-the-same-OPU variances are shaded at corresponding Raman shift ranges. All Raman spectra are shaded with a range of three times of the standard error vertically.


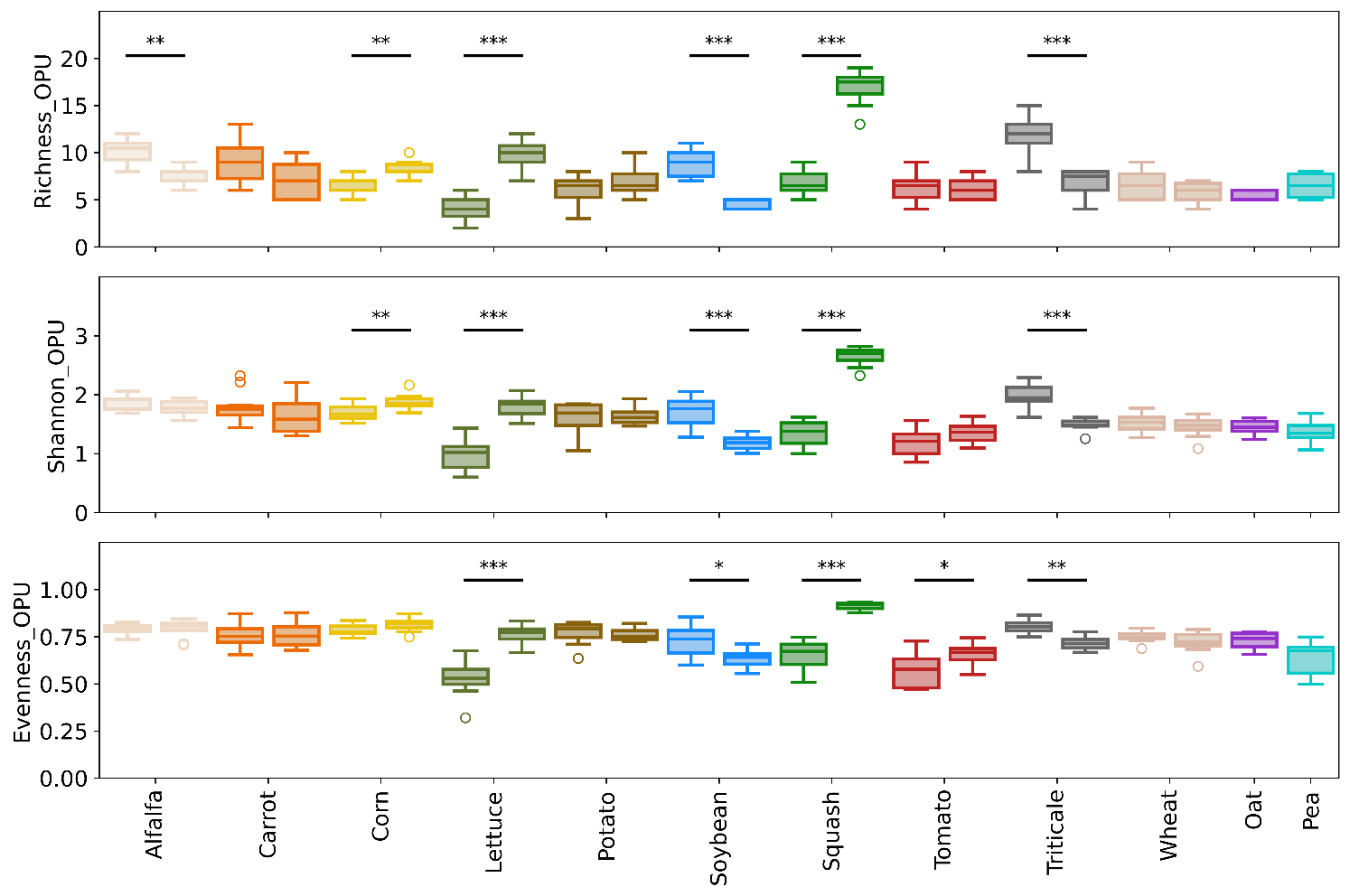


**Supplementary Figure 7.** Distribution of richness, Shannon’s diversity index, and evenness of OPUs. Paired results for the same crop from conventional (left) and organic (right) farms were displayed side by side. Significant differences were identified using Mann-Whitney U test and indicated by asterisks: *: *p* < 0.05; **: *p* < 0.01; and ***: *p* < 0.001.


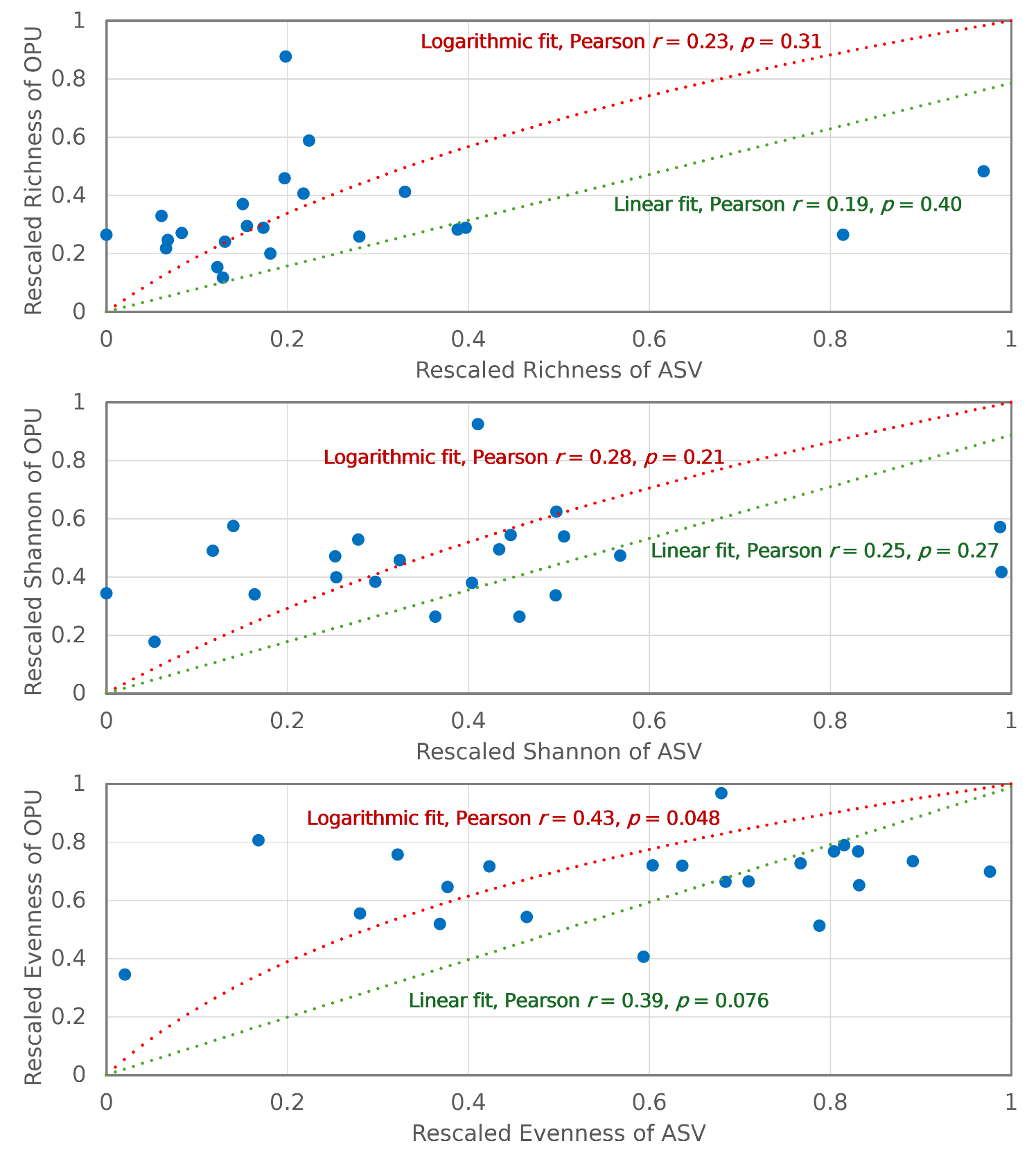


**Supplementary Figure 8.** Logarithmic and linear fits of richness, Shannon’s diversity index, and evenness between OPUs and ASVs.
